# Integrated assessment of growth performance, carcass characteristics, and economic efficiency of three broiler strains raised under humid-tropical conditions

**DOI:** 10.1371/journal.pone.0351927

**Published:** 2026-06-22

**Authors:** Dennis Owusu Acheampong, Kwaku Adomako, Bismark Kyei

**Affiliations:** Department of Animal Science, Faculty of Agriculture, Kwame Nkrumah University of Science and Technology, Kumasi, Ghana; Ain Shams University Faculty of Agriculture, EGYPT

## Abstract

This study evaluated the growth performance, carcass characteristics, economic efficiency, health status, and growth patterns of three commercial broiler strains (Hubbard, Cobb 500, and Ross 308) reared under humid tropical conditions in Ghana. A total of 300 day-old chicks were allocated to a completely randomized design with five replicate pens per strain and reared for six weeks under uniform management conditions. Performance traits, carcass and internal organ characteristics, mortality, and morbidity were assessed, and growth patterns were modeled using the Gompertz function. Although initial body weights differed among strains, no significant differences were observed in final body weight, weight gain, feed intake, feed conversion ratio, or feed cost per kg weight gain. Carcass characteristics were also comparable across strains. Cobb 500 exhibited numerically higher growth rate and asymptotic weight based on the Gompertz model, whereas Ross 308 showed slower growth but lower mortality and greater development of digestive organs, suggesting improved adaptability to tropical conditions. Overall, the findings indicate that while the three broiler strains demonstrate similar production performance under tropical conditions, differences in growth dynamics and survivability may influence strain suitability. These results highlight the importance of balancing growth efficiency with environmental adaptability when selecting broiler genotypes for humid tropical production systems.

## Introduction

The poultry industry plays a vital role in global food security and nutrition by providing a sustainable supply of high-quality protein through meat and eggs. Among all agricultural commodities, poultry remains the fastest-growing livestock sector in terms of production. With the continuous rise in the global population, demand for poultry products continues to increase due to their cost-effectiveness, accessibility, and widespread acceptance as a reliable protein source [1]. Over the past decade, broiler chicken production has expanded significantly compared with other poultry species, driven by increasing consumer demand for affordable and nutritious protein sources [2]. As major contributors to the global poultry sector, broiler chickens are highly valued for their rapid growth rates, exceptional feed conversion efficiency, and adaptability to diverse production systems [3].

Poultry production in tropical environments is frequently constrained by high ambient temperatures, elevated humidity, and fluctuating management conditions, which can negatively affect broiler growth performance, feed efficiency, and welfare. These environmental challenges are particularly pronounced in hot-humid production systems where birds experience thermal stress that can limit productivity [4].

In Ghana, the poultry sector forms an important component of the agricultural economy, contributing to employment, food security, and income generation. Despite its importance, the industry faces substantial challenges including high production costs, disease susceptibility, and limited access to quality feed resources [5]. Poultry production in Ghana occurs under humid climatic conditions characterized by high ambient temperatures ranging from 28–34 °C and relative humidity frequently exceeding 70%. In many commercial poultry farms, housing systems are typically open-sided with natural ventilation, which exposes birds to greater fluctuations in environmental temperature and humidity. These management conditions differ markedly from intensive climate-controlled systems commonly used in temperate regions and can influence broiler productivity and economic returns [6–8].

To address production constraints and improve efficiency, breeders and geneticists have focused on developing broiler strains that exhibit rapid growth, improved disease resistance, and efficient feed utilization. Continuous genetic selection, coupled with advances in nutrition and management practices, has markedly enhanced broiler growth performance and reduced the number of days required to reach market weight [9]. Commercial broiler strains such as Cobb 500, Ross 308, Arbor Acres, and Hubbard are widely recognized for their rapid growth rates and ability to achieve high market weights within a short production cycle, thereby improving productivity and economic efficiency.

Although intensive genetic selection for increased body weight and accelerated growth has greatly improved production efficiency, it has also increased the susceptibility of modern broilers to environmental challenges. High ambient temperatures typical of warm production environments impose significant thermal stress on birds and remain a major limitation to poultry production [10,11]. Broiler chickens bred for rapid growth and high meat yield rely heavily on favorable environmental conditions to fully express their genetic potential. Exposure to heat stress has been widely associated with reduced feed intake, impaired growth performance, compromised immune function, and decreased carcass yield in broiler chickens [3,4,12]. Consequently, effective management of high environmental temperatures is essential for maintaining optimal production efficiency and ensuring quality meat output. Supporting this, [13] demonstrated that exposure to elevated ambient temperatures significantly diminished the performance of broiler chickens, further highlighting the detrimental effects of heat stress on production outcomes [14].

Despite the introduction of several commercial broiler strains into Ghana’s poultry production systems, comprehensive comparative evaluations of their performance remain limited. In particular, the relative growth performance and adaptability of Hubbard, Cobb 500, and Ross 308 broilers under Ghana’s warm climatic conditions are not fully understood. Therefore, this study was designed to assess the influence of genotype on growth performance, carcass characteristics, feed conversion ratio, morbidity, and mortality of these three broiler strains reared in a tropical production environment. In addition, the Gompertz growth model was employed to characterize the growth patterns of the three broiler strains raised under these conditions.

## Materials and methods

### Ethics statement

All experimental procedures were conducted in accordance with institutional guidelines for animal care and were approved by the Animal Ethics Committee of Kwame Nkrumah University of Science and Technology (Approval No: KNUST-0018).

### Animal welfare considerations and humane endpoints

Birds were monitored daily for health status, behavior, feed and water intake, and general activity throughout the six-week experimental period. Humane endpoints were predefined to minimize potential suffering. Birds were to be removed from the experiment if they exhibited severe clinical signs such as persistent inability to stand or walk, severe lethargy, respiratory distress, unresponsiveness to stimuli, prolonged refusal to feed or drink (>24 hours), or rapid body weight loss exceeding 20% relative to age-matched pen averages.

All animal handling and husbandry procedures were conducted by trained personnel in accordance with institutional animal welfare and ethical guidelines. Efforts were made to minimize stress, pain, and discomfort through appropriate housing, ventilation, nutrition, biosecurity measures, and routine health monitoring. No invasive procedures were conducted during the experimental period; therefore, analgesics or anesthetics were not required prior to routine slaughter for carcass evaluation at day 42.

A total of 300 broiler chickens were used in the study. None of the birds exhibited severe or prolonged clinical signs that met the predefined humane endpoint criteria, and therefore no birds required euthanasia during the experimental period.

Six birds (2% of the total population) died naturally during the study, primarily within the first two weeks of brooding. These mortalities occurred before the birds reached humane endpoint criteria and were attributed mainly to early brooding stress and environmental adaptation challenges commonly observed in commercial broiler production under tropical conditions. All mortalities were recorded daily and included in the analysis.

### Experimental site and duration

The study was conducted at the Poultry Section of the Department of Animal Science, Kwame Nkrumah University of Science and Technology (KNUST), Kumasi, Ghana. The Department of Animal Science lies between longitude 01º 033’ W and latitude 06º 41’ N in a hot humid environment. Annually, the area receives rainfall of about 1400 mm to 1700 mm, with annual temperature and relative humidity of 25°C – 35°C and 74% − 85% respectively. The study lasted for six (6) weeks; from 11^th^ November, 2024–22^nd^ December, 2024.

### Experimental design

A completely randomized design (CRD) was employed using three broiler strains (Hubbard, Cobb 500, and Ross 308). A total of 300 day-old chicks were randomly allocated to the three strains, with five replicate pens per strain and 20 birds per pen.

The pen (replicate) was considered the experimental unit for growth performance variables, including body weight, feed intake, and feed conversion ratio, as birds were group-housed and managed collectively within each pen.

Individual birds were considered the observational units for carcass and internal organ measurements. Birds were not sex-separated during the experimental period to simulate commercial production conditions. However, for carcass evaluation, both male and female birds were randomly selected from each strain to ensure representative sampling.

### Selection of strains

The study examined three commercial broiler chicken strains: Strain A (Hubbard), Strain B (Cobb 500), and Strain C (Ross 308). These strains were chosen for their commercial viability, growth potential, and genetic diversity, being predominant in Ghana’s poultry farming. Day-old chicks (DOC) from reputable hatcheries were used, with initial average weights of 0.036 kg for Strain A, 0.043 kg for Strain B, and 0.043 kg for Strain C.

### Housing and management practices

The chicks were brooded in replicated pens for the first two weeks, using 100-watt incandescent bulbs for optimal temperatures. They received glucose and water upon arrival to alleviate transportation stress. The housing utilized deep litter bedding of wood shavings, and the birds were exposed to 24-hour lighting during brooding. Strict biosecurity measures were enforced, including footbaths, restricted access, and routine vaccinations. Prior to the study, the poultry house, water troughs, and feeders were thoroughly cleaned, disinfected, and dried.

### Feed and ingredients composition

The study involved feeding birds a four-phase diet consisting of pre-starter, starter, grower, and finisher feeds over six weeks. Koudijs Galdus Pre-starter was provided in the first week (Table 1), starter feed in the second and third weeks, grower feed in the fourth and fifth weeks, and finisher feed in the sixth week (Table 2). Birds had *ad libitum* access to feed and water throughout the study, with weekly recordings of feed intake (FI).

**Table 1:** KBC 35% Nutritional Content Calculated and Ingredients.

Ingredients	Value	Unit
Crude Protein	43.00	%
Crude Fat	2.93	%
Lysine	2.95	%
Methionine	1.28	%
Calcium	2.40	%
Phosphorus	0.93	%
M. Energy	2,263	Kcal

Ingredients: Corn, Soymeal, Wheat by-products, Minerals, premixes and Soya Oil. KBC 35% = Koudijs Broiler Concentrate (35%)

**Table 2:** Feed Ingredients and Composition for Starter, Grower, and Finisher Diets.

Ingredient	Starter (%)	Grower (%)	Finisher (%)
KBC 35%	35.00	30.00	25.00
Maize	65.00	70.00	75.00
Toxin Binder (kg)	0.50	0.50	0.50
Total	100	100	100
M. Energy	2,937	2,989	3,041

KBC 35% = Koudijs Broiler Concentrate (35%), used across all feeding phases (starter, grower, and finisher) in varying proportions. Toxin binder is added at 0.50 kg per feed type.

### Data collection

#### Growth performance parameters.

The body weight (BW) and feed intake (FI) were recorded weekly. Daily feed intake (DFI) and daily weight gain (DWG) were derived from weekly feed intake (WFI) and weekly weight gain (WWG) measurements. The feed conversion ratio (FCR) was calculated as the ratio of feed intake to body weight gain (BWG).

### Carcass and internal organ traits measurements

At day 42, five birds per strain (2 males and 3 females) with body weights closest to the treatment mean were selected to represent the average performance of each group. The selected birds were subjected to a 12-hour feed withdrawal period with access to water before slaughter for carcass and internal organ assessment. Although the sample size was relatively small compared with the total experimental population, selecting birds with body weights close to the treatment mean is a common approach in broiler carcass studies to ensure that sampled individuals accurately represent the average performance of each treatment group and to minimize sampling bias [15,16].

### Economic efficiency metrics

The feed conversion ratio and feed cost per kg of weight gain (in Ghana Cedis, GHS) were used as indices to assess economic efficiency.


$$\begin{array}{c} \text{FCR}=\frac{\text{Total Feed Intake }(\text{kg})}{\text{Total Body Weight Gain }(\text{kg})} \end{array}$$

$$\begin{array}{c} \text{Feed Cost}/\text{kg Gain as}:\;\frac{\text{Total Feed Consumed }(\text{kg})\times \text{Cost of Feed }(\text{GHS}/\text{kg})}{\text{Total Body Weight Gain }(\text{kg})} \end{array}$$

### Mortality and morbidity records

Mortality and morbidity data were recorded throughout the 42-day experimental period for all broiler strains (Hubbard, Cobb 500, and Ross 308). Birds were managed under uniform housing, feeding, and environmental conditions to ensure that observed differences were attributable to strain effects rather than management variability.

Mortality was recorded daily for each replicate pen. Dead birds were removed promptly upon observation and the number of mortalities per replicate was documented. For analysis, mortality counts were aggregated at specific age intervals (days 1, 7, 14, 21, 28, 35, and 42), corresponding to key production stages. These counts were expressed as the number of deaths per replicate at each age interval and used to evaluate both total mortality per strain and temporal mortality patterns across the production cycle.

Morbidity was assessed through routine daily observation of birds for clinical signs of illness, including lethargy, reduced feed intake, abnormal posture, respiratory distress, or other visible indicators of poor health. Birds exhibiting any observable clinical symptoms were classified as morbid.

Morbidity was recorded as a binary outcome at the replicate level (0 = absence of morbidity, 1 = presence of morbidity) at the same age intervals used for mortality assessment (days 1, 7, 14, 21, 28, 35, and 42). This approach allowed for consistent tracking of disease occurrence over time while minimizing observer bias.

For both mortality and morbidity, data were compiled across five replicates per strain. Mortality data were treated as count variables, while morbidity data were treated as binary outcomes. These datasets were subsequently used to evaluate overall strain differences and age-related trends in health status.

### Analytical framework

#### Growth curve modeling using the Gompertz function.

The growth trajectory of broiler chickens was modeled using the nonlinear Gompertz growth function, which is widely accepted for describing sigmoidal growth patterns in poultry species [17,18]. The model is expressed as:


$$\begin{array}{c} \text{W}(\text{t})\;=\text{ A }\times \text{ exp}(-\text{B }\times \text{ exp}(-\text{k }\times \text{ t})) \end{array}$$

where W(t) is the body weight at age t (days), A represents the asymptotic mature body weight (kg), B is a scaling constant related to initial body weight, and k is the growth rate constant.

The model was fitted using nonlinear least squares (nls) in R (version 4.4.1). Initial parameter values (A = 3, B = 2, k = 0.1) were used to ensure model convergence. Weekly body weight and age data were used for model fitting.

To account for biological variation while maintaining stable parameter estimation, data were pooled within strains across replicates, and a separate model were fitted for each strain (Hubbard, Cobb 500, and Ross 308). This approach allowed the estimation of strain-specific growth curves representing overall production performance under uniform management conditions.

Model performance was evaluated using coefficient of determination (pseudo-R²) and root mean square error (RMSE). These statistics were computed by comparing observed and fitted values from each strain-specific model. In addition, Akaike Information Criterion (AIC) was used to assess relative model adequacy across strains.

To address between-strain differences in growth dynamics, Gompertz parameters (A, B, and k) were compared descriptively, and 95% confidence intervals of parameter estimates were generated using the delta method/ asymptotic standard errors from the nls output. This allowed inference on differences in mature weight and growth rate among strains.

Graphical assessment was performed using ggplot2, where observed data points were plotted against fitted Gompertz curves for each strain, providing visual validation of model fit and growth pattern differences.

### Corelation analysis

Pearson’s correlation analysis was used to evaluate the relationships among carcass traits in the three broiler strains. The analysis was conducted in R software (version 4.4.1; R Core Team, Vienna, Austria). Only quantitative carcass variables, including live body weight measurements, carcass cut weights, dressing percentage, and abdominal fat, were included in the analysis, while non-biological variables such as replicate were excluded.

Correlation coefficients (r) were computed using the Hmisc package in R, which also provided associated probability values (p-values) for testing statistical significance. Prior to analysis, data were structured to retain only numeric variables relevant to carcass performance. Pearson’s correlation was selected due to its suitability for assessing linear relationships between continuous traits.

The strength and direction of associations were interpreted based on correlation coefficients, where positive values indicate direct relationships and negative values indicate inverse relationships between traits. Significance levels were considered at p < 0.05, < 0.01, and < 0.001. The resulting correlation matrix was summarized in tabular form and visualized using a heatmap generated in the ggplot2 package to enhance interpretation of trait interrelationships.

Graphical visualization of the correlation matrix was used to facilitate interpretation of trait clustering and identify strongly associated carcass characteristics.

### Statistical analysis

All statistical analyses were performed using R software (version 4.4.1; R Core Team, Vienna, Austria). Data cleaning, transformation, and analysis were conducted using the packages dplyr, tidyr, ggplot2, broom, car, MASS, and minpack.lm.

Growth performance, feed efficiency, economic traits, carcass characteristics, and internal organ weights were analysed using one-way analysis of variance (ANOVA) to test the effect of broiler strain (Hubbard, Cobb 500, and Ross 308). For traits measured at market age (42 days), strain was considered the main fixed effect.

The general statistical model was:


$$\begin{array}{c} {\text{Y}}_{\text{ij}}=\mu +{\text{S}}_{\text{i}}+\epsilon_{\text{ij}} \end{array}$$

where Yᵢⱼ is the observed trait, μ is the overall mean, Sᵢ is the fixed effect of strain, and εᵢⱼ is the random error term.

For traits where initial body weight was considered a potential covariate (e.g., growth-related performance variables), analysis of covariance (ANCOVA) was applied using initial body weight as a covariate to adjust for early variation among experimental units. Homogeneity of regression slopes was assumed prior to ANCOVA interpretation.

For all ANOVA and ANCOVA models, means were estimated for each strain, and variability was expressed as standard error of the mean (SEM). Statistical significance was declared at p < 0.05.

Mortality and morbidity data were analysed as count and proportion variables. Total mortality and age-specific trends were first summarised descriptively by strain. To evaluate the effect of strain and age on mortality, a negative binomial regression model (glm.nb) was fitted to account for overdispersion in count data.

The model included main effects of strain and age, as well as their interaction:


$$\begin{array}{c} \text{log}(\mu )\;=\;\beta_{0}\;+\;\beta_{\text{1}}\text{ Strain }+\;\beta_{\text{2}}\text{ Age }+\;\beta_{\text{3}}(\text{Strain }\times \text{ Age}) \end{array}$$

Model outputs were presented as incidence rate ratios (IRR) with corresponding 95% confidence intervals.

Morbidity, recorded as a binary outcome (0 = absence, 1 = presence), was initially analysed using a binomial generalized linear model (GLM) with a logit link function:


$$\begin{array}{c} \text{logit}(\text{p})\;=\;\beta_{0}\;+\;\beta_{\text{1}}\text{ Strain }+\;\beta_{\text{2}}\text{ Age }+\;\beta_{\text{3}}(\text{Strain }\times \text{ Age}) \end{array}$$

where strain was included as a categorical factor and age as a continuous covariate. Predicted probabilities were generated from the fitted model to visualise strain-specific morbidity trends across age.

However, due to the extremely low number of morbidity events and the absence of cases in two of the three strains, the data exhibited quasi-complete separation, resulting in unstable parameter estimates and unreliable confidence intervals. Consequently, morbidity results were interpreted descriptively, and model outputs were not used for inferential conclusions.

Growth trajectories of broiler strains were modeled using the Gompertz function as described above in analytical framework.

All results were presented as means ± SEM. Graphical outputs, including growth curves, mortality and morbidity trends, and performance comparisons, were generated using the ggplot2 package. Predicted values from fitted models were overlaid on observed data to assess model fit visually.

### Physiological measurements (Body temperature)

Body temperature of broiler chickens was measured weekly using a digital thermometer at three periods of the day: morning, afternoon, and evening. Measurements were taken for each strain throughout the six-week experimental period. Weekly mean body temperature values for Strain A, B, and C are presented in Table 3.

**Table 3:** Mean Body Temperature (°C) of Strain A, B, and C Broilers.

Week	Period	Strain A (°C)	Strain B (°C)	Strain C (°C)
Week 1	Morning	40.85	40.80	40.70
	Afternoon	41.15	41.10	41.00
	Evening	41.05	41.00	40.90
Week 2	Morning	40.95	40.90	40.80
	Afternoon	41.25	41.20	41.10
	Evening	41.15	41.10	41.00
Week 3	Morning	41.05	41.00	40.90
	Afternoon	41.35	41.30	41.20
	Evening	41.25	41.20	41.10
Week 4	Morning	41.15	41.10	41.00
	Afternoon	41.45	41.40	41.30
	Evening	41.35	41.30	41.20
Week 5	Morning	41.35	41.30	41.20
	Afternoon	41.65	41.60	41.50
	Evening	41.55	41.50	41.40
Week 6	Morning	41.45	41.40	41.30
	Afternoon	41.75	41.70	41.60
	Evening	41.65	41.60	41.50

### Environmental conditions during the study

Ambient environmental conditions were monitored throughout the study period. Ambient temperature and relative humidity were recorded daily using a digital thermo-hygrometer placed inside the poultry house, and weekly means were calculated. The mean weekly ambient temperature and relative humidity during the six-week period are presented in Table 4.

**Table 4:** Mean Ambient Temperature (°C) and Relative Humidity (%) Recorded at the Animal Science Department, KNUST.

Week	Parameters	Mean
Week 1	Ambient Temperature	26.83
	Relative Humidity	76.0
Week 2	Ambient Temperature	27.33
	Relative Humidity	75.0
Week 3	Ambient Temperature	27.63
	Relative Humidity	74.7
Week 4	Ambient Temperature	28.0
	Relative Humidity	73.7
Week 5	Ambient Temperature	28.37
	Relative Humidity	73.0
Week 6	Ambient Temperature	28.67
	Relative Humidity	74.7

## Results

### Growth performance and economic efficiency of the three broiler strains

The growth performance parameters of the experimental birds are summarized in Table 5. Analysis of the initial body weight of the day-old chicks revealed a significant difference (p < 0.05) between Strain A and the other strains, with Strain A exhibiting a notably lower mean weight (0.036 kg) compared to Strain B (0.043 kg) and Strain C (0.043 kg). However, no significant differences (p > 0.05) were observed among the three strains in terms of weight gain and final body weight at 42 days of age. Numerically, Strain B recorded the highest weight gain and final body weight, followed by Strain A and C.

**Table 5:** Growth performance of Strain A, Strain B, and Strain C.

Parameter	Strain A	Strain B	Strain C	SEM	P-value
Initial Weight (kg)	0.036^b^	0.043^a^	0.043^a^	0.000489	0.001
Weight Gain (kg)	2.502	2.572	2.457	0.064924	0.477
Final Body Weight (kg)	2.539	2.615	2.500	0.064947	0.467
Feed Conversion Ratio (FCR)	1.674	1.754	1.668	0.030673	0.172
Daily Feed Intake (kg)	0.099	0.106	0.098	0.002259	0.060
Daily Weight Gain (kg)	0.059	0.061	0.059	0.001521	0.472
Weekly Feed Intake (kg)	0.694	0.741	0.683	0.015684	0.075
Weekly Weight Gain (kg)	0.417	0.429	0.410	0.010848	0.480
Final Feed Intake (kg)	4.169	4.467	4.098	0.092977	0.054
Cost of Feed/kg	10.08	10.08	10.08	0.000000	0.873
Feed Cost/kg Weight Gain (GHS)	16.83	17.16	16.54	0.417017	0.620

^a-b^ Means with different superscripts on the same row are significantly different at p < 0.05.

To consider feed conversion ratio, we noticed that although Strains A and C (1.674 and 1.668, respectively) were slightly better than Strain B (1.754), the differences were not statistically significant (p > 0.05). Similarly, no significant differences (p > 0.05) were observed in daily, weekly, or final feed intake among the strains. Nonetheless, Strain B consistently demonstrated higher values for all feed intake parameters, followed by Strains A and C. This trend suggests that Strain B tended to consume more feed, even though the variations were not statistically significant (p > 0.05). Furthermore, the cost of feed per kg and the feed cost per kg of weight gain did not differ significantly (p > 0.05) among the strains.

### Carcass traits analysis of the three strains

As presented in Table 6, our analysis revealed no significant differences (p > 0.05) in carcass parameters such as bled weight, de-feathered weight, dressed weight, or dressing percentage among Strains A, B, and C. Similarly, breast weight did not differ significantly (p > 0.05) between the strains, although Strain B (0.768 kg) and C (0.767 kg) recorded higher values compared to Strain A (0.745 kg).

**Table 6:** Carcass traits of Strain A, Strain B, and Strain C.

Parameter	Strain A	Strain B	Strain C	SEM	P-value
Bled Weight (kg)	2.450	2.544	2.449	0.052	0.399
De-feathered Weight (kg)	2.360	2.437	2.385	0.048	0.577
Dressed Weight (kg)	2.180	2.244	2.141	0.035	0.206
Dressing Percentage (%)	85.10	83.86	86.32	1.076	0.377
Breast Weight (kg)	0.745	0.768	0.767	0.024	0.754
Shank Weight (kg)	0.080	0.082	0.074	0.005	0.529
Neck Weight (kg)	0.125	0.12	0.125	0.007	0.890
Head Weight (kg)	0.053	0.05	0.050	0.003	0.695
Wings Weight (kg)	0.204	0.211	0.182	0.007	0.051
Thighs and Drumsticks (kg)	0.500	0.551	0.495	0.016	0.097
Abdominal Fat (kg)	0.031	0.022	0.028	0.006	0.530

The weights of the shank, neck, and head were also statistically similar (p > 0.05) across the three strains. No significant differences (p > 0.05) were observed in wing weight and thigh-drumstick weight among the strains. Although Strain C showed numerically lower values for these parameters compared to Strains A and B, the variations were not statistically significant. Likewise, abdominal fat weight did not differ significantly (p > 0.05) among the strains.

### Internal organ traits analysis

Significant differences (p < 0.05) were observed in full intestine weight, empty intestine weight, and liver weight, with Strain C recording the highest values for all three parameters, as presented in Table 7. In contrast, no significant differences (p > 0.05) were detected among the strains for gizzard weight, heart weight, and spleen weight, indicating comparable development of these organs across the groups.

**Table 7:** Internal organ traits of Strain A, Strain B, and Strain C.

Parameter	Strain A	Strain B	Strain C	SEM	P-value
Full Intestine (g)	98^b^	110^ab^	125^a^	5.673	0.031
Empty Intestine (g)	55^b^	60^ab^	69^a^	3.316	0.035
Full Gizzard (g)	69	65	62	4.444	0.556
Empty Gizzard (g)	44	42	40	2.629	0.600
Heart (g)	11	10	9	0.667	0.262
Liver (g)	52^b^	46^ab^	60^a^	3.231	0.048
Spleen (g)	5	5	6.4	0.374	0.251

^a, b, ab^ Means with different superscripts on the same row are significantly different at p < 0.05.

### Gompertz growth curve of the strains

To evaluate and compare the growth patterns among the strains, the Gompertz growth model was applied. As shown in Table 8, Strain B exhibited the highest asymptotic weight (4.30 kg), followed by Strain A (4.10 kg) and Strain C (3.90 kg). The growth rate constant (k) was also greatest in Strain B (0.057), suggesting a faster growth potential relative to Strain A (0.054) and C (0.050).

**Table 8:** Gompertz growth curve parameters for Strain A, B, and C.

Strain	Asymptotic Weight (*A*) (kg)	Growth Rate (*k*) (per day)	Scaling Parameter (*B*)
B	4.30 ± 0.12	0.057 ± 0.003	4.52 ± 0.15
A	4.10 ± 0.10	0.054 ± 0.002	4.36 ± 0.12
C	3.90 ± 0.09	0.050 ± 0.002	4.21 ± 0.10

**Fig 1:**
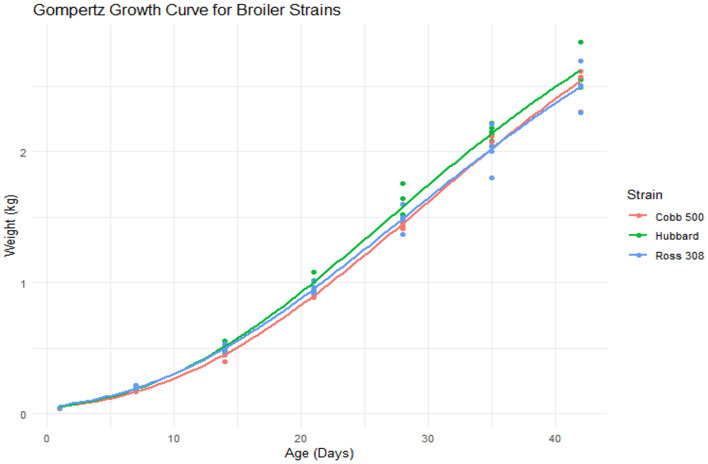
Gompertz growth curves showing the growth patterns among the strains.

Fig 1 shows clear separation among the fitted Gompertz growth curves for the three strains across the rearing period. Strain B consistently plots above Strains A and C, reflecting its greater asymptotic weight and higher overall growth rate. The curves also show that the inflection point representing the phase of maximum relative growth occurs earliest in Strain B at approximately 26.5 days, followed by Strain A at 27.3 days and Strain C at 28.7 days. At these respective inflection points, the predicted body weights are approximately 1.58 kg for Strain B, 1.51 kg for Strain A, and 1.43 kg for Strain C. Collectively, these results demonstrate that Strain B attains higher predicted weights more rapidly, whereas Strain C exhibits the slowest growth and approaches the lowest asymptotic weight.

### Correlations among traits

**Fig 2:**
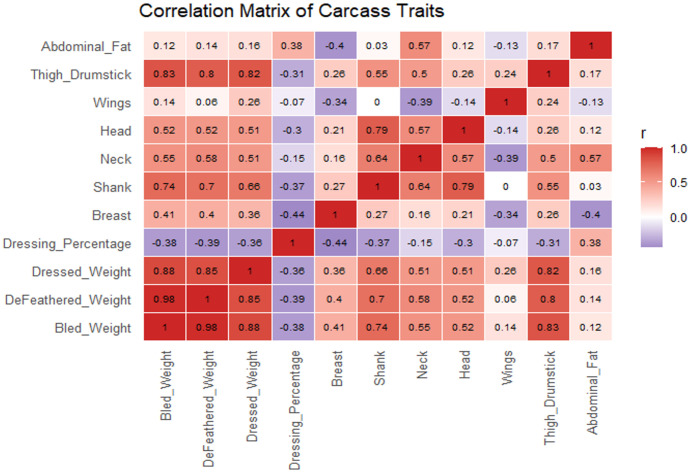
Correlation heatmap showing the relationships among growth and carcass traits of the three broiler strains.

As presented in Table 9 and Fig 2, a very strong positive correlation was observed between bled weight and de-feathered weight (r = 0.98, p < 0.001), as well as between bled weight and dressed weight (r = 0.88, p < 0.001). Similarly, de-feathered weight was strongly correlated with dressed weight (r = 0.85, p < 0.001). These relationships indicate a high level of consistency among the primary carcass weight measurements, suggesting that increases in live processing weights are directly reflected in final carcass yield.

**Table 9:** Correlations among growth, carcass, and internal organ traits.

	Bled_Weight	DeFeathered_Weight	Dressed_Weight	Dressing_Percentage	Breast	Shank	Neck	Head	Wings	Thigh_Drumstick	Abdominal_Fat
**Bled_Weight**	—	0.98***	0.88***	−0.38	0.41	0.74**	0.55*	0.52*	0.14	0.83***	0.12
**DeFeathered_Weight**	0.98***	—	0.85***	−0.39	0.40	0.70**	0.58*	0.52*	0.06	0.80***	0.14
**Dressed_Weight**	0.88***	0.85***	—	−0.36	0.36	0.66**	0.51	0.51	0.26	0.82***	0.16
**Dressing_Percentage**	−0.38	−0.39	−0.36	—	−0.44	−0.37	−0.15	−0.30	−0.07	−0.31	0.38
**Breast**	0.41	0.40	0.36	−0.44	—	0.27	0.16	0.21	−0.34	0.26	−0.40
**Shank**	0.74**	0.70**	0.66**	−0.37	0.27	—	0.64*	0.79***	0.00	0.55*	0.03
**Neck**	0.55*	0.58*	0.51	−0.15	0.16	0.64*	—	0.57*	−0.39	0.50	0.57*
**Head**	0.52*	0.52*	0.51	−0.30	0.21	0.79***	0.57*	—	−0.14	0.26	0.12
**Wings**	0.14	0.06	0.26	−0.07	−0.34	0.00	−0.39	−0.14	—	0.24	−0.13
**Thigh_Drumstick**	0.83***	0.80***	0.82***	−0.31	0.26	0.55*	0.50	0.26	0.24	—	0.17
**Abdominal_Fat**	0.12	0.14	0.16	0.38	−0.40	0.03	0.57*	0.12	−0.13	0.17	—

Pearson correlation coefficients (r) are presented. Significance levels are denoted as * p < 0.05, ** p < 0.01, and *** p < 0.001.

Among carcass cuts, thigh and drumstick weight showed strong positive associations with bled weight (r = 0.83, p < 0.001), de-feathered weight (r = 0.80, p < 0.001), and dressed weight (r = 0.82, p < 0.001). This suggests that heavier birds tend to allocate more mass to the lower body portions, highlighting the importance of these cuts in overall carcass composition.

Moderate positive correlations were also observed between bled weight and shank weight (r = 0.74, p < 0.01), as well as de-feathered weight and shank weight (r = 0.70, p < 0.01), indicating that skeletal development increases proportionally with overall body weight. In addition, neck weight and head weight exhibited moderate correlations with the primary carcass weights (r = 0.51–0.58), further supporting coordinated growth across anatomical components.

Interestingly, dressing percentage showed weak and non-significant negative correlations with most carcass weight traits (r = −0.36 to −0.39), suggesting that higher live or processed weights do not necessarily translate into improved dressing efficiency. This implies that proportional yield may be influenced more by body composition than by absolute weight.

The breast weight, a key economic trait, exhibited weak positive correlations with carcass weights (r = 0.36–0.41), but these relationships were not statistically significant, indicating variability in breast muscle deposition independent of overall body weight.

Abdominal fat showed generally weak and non-significant relationships with most carcass traits, except for a moderate positive correlation with neck weight (r = 0.57, p < 0.05). This suggests that fat deposition may not be strongly linked to general carcass growth but could be associated with specific anatomical regions.

Overall, the correlation structure demonstrates that carcass weight traits are strongly interrelated, while cut yields and fat deposition exhibit more variable and trait-specific relationships.

Positive correlations indicate that as one trait increases, the other tends to increase, whereas negative correlations show inverse relationships. The intensity of the colour represents the strength of the correlation, with darker shades indicating stronger associations.

### Mortality

**Fig 3:**
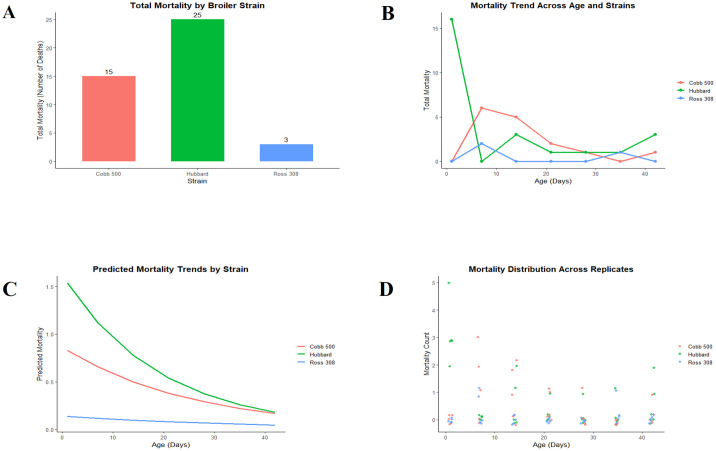
Mortality trends observed among the three broiler strains during the experimental period. (A) Total mortality by broiler strain. (B) Mortality trend by age and strains. (C) Predicted mortality trend by strain. (D) Mortality distribution across replicates. The figures depict the variation in mortality rates over the six-week rearing period for each strain.

Fig 3A shows total mortality varied across broiler strains. Strain A recorded the highest mortality (25 deaths), followed by Strain B (15 deaths), while Strain C exhibited the lowest mortality (3 deaths). These results indicate substantial variation in survivability among the strains under the same management conditions.

Mortality patterns differed across age and strain as seen in Fig 3B. Strain A exhibited a pronounced early mortality peak at day 1, accounting for the majority of deaths within this strain. In contrast, Strain B showed moderate mortality primarily concentrated between days 7 and 14, after which mortality declined markedly. Strain C maintained consistently low mortality throughout the production period, with only sporadic cases observed. Overall, mortality tended to decrease with increasing age across all strains, although the magnitude and timing of mortality differed among strains.

Replicate-level variation in mortality is shown in Fig 3D. While some variability was observed within strains, particularly in Strain A, the overall distribution supports the higher mortality burden in this strain relative to Strain B and Strain C.

As presented in Table 10 below, the effects of strain, age, and their interaction on mortality were evaluated using a negative binomial regression model. No statistically significant effects were observed at the 5% level (p > 0.05); however, marginal trends were evident. Hubbard showed a higher mortality rate compared to Strain B (IRR = 1.88, 95% CI: 0.55–6.46, p = 0.318), although this difference was not statistically significant. In contrast, Strain C exhibited a lower mortality rate relative to Strain B (IRR = 0.16, 95% CI: 0.02–1.33), with a marginal level of significance (p = 0.090), suggesting improved survivability.

**Table 10:** Negative binomial regression analysis of mortality in broiler strains across age.

Variable	IRR	95% CI	p-value
Intercept	0.86	0.34 - 2.20	0.755
Strain A (vs Strain B)	1.88	0.55 - 6.46	0.318
Strain C (vs Strain B)	0.16	0.02 - 1.33	0.090
Age (days)	0.96	0.92 - 1.01	0.097
Strain A × Age	0.99	0.93 - 1.05	0.672
Strain C × Age	1.01	0.92 - 1.12	0.803

IRR = Incidence Rate Ratio; CI = Confidence Interval;

Reference category = Cobb 500; Model: Negative binomial regression

Significance level: p < 0.05

Age was associated with a slight reduction in mortality risk (IRR = 0.96, 95% CI: 0.92–1.01, p = 0.097), indicating a general decline in mortality as birds aged. The interaction between strain and age was not statistically significant, suggesting that mortality trends over time were broadly similar across strains.

Model-based predictions further illustrate these patterns (Fig 3C). Predicted mortality declined with increasing age across all strains, with Strain A maintaining consistently higher predicted mortality levels, while Strain C exhibited the lowest predicted mortality throughout the production cycle.

### Morbidity

**Fig 4:**
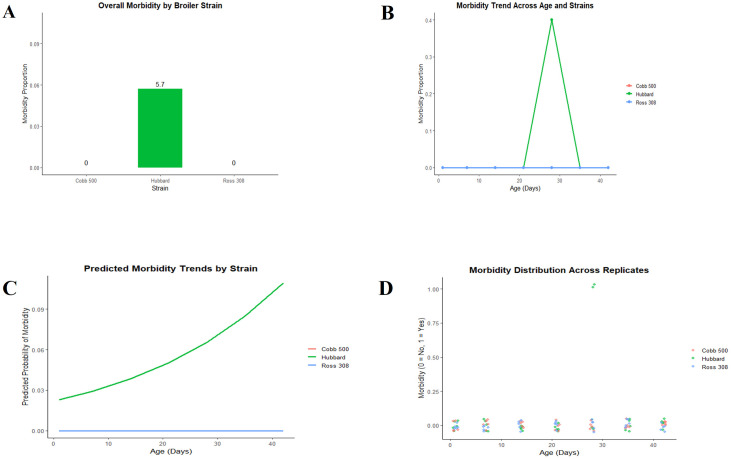
Morbidity trends observed among the three broiler strains during the experimental period. (A) Overall morbidity by broiler strain. (B) Morbidity trend across age and strain. (C) Predicted morbidity trends by strains. (D) Morbidity distribution across replicates. The figures depict the variation in morbidity rates over the six-week rearing period for each strain.

Morbidity incidence was low across all broiler strains (Fig 4A). Only the Strain A recorded morbidity cases, with a total of 2 cases corresponding to a prevalence of 5.71%, while no morbidity was observed in Strain B or Strain C throughout the study period as seen in Table 11.

**Table 11:** Overall morbidity by broiler strain.

Strain	Total Cases	Observations	Prevalence (%)
Strain B	0	35	0.0
Strain A	2	35	5.71
Strain C	0	35	0.0

Prevalence calculated as proportion of morbidity cases per total observations

Morbidity defined as presence (1) or absence (0) of disease

Morbidity patterns across age and strain are presented in Fig 4B. Morbidity events were rare and occurred only at a single time point (day 28) in the Hubbard strain. No morbidity was recorded at any age in Strain B and Strain C. Consequently, no consistent age-related pattern of morbidity was observed across the production cycle.

Replicate-level variation in morbidity is shown in Fig 4D. The distribution confirms the sporadic nature of morbidity, with only isolated occurrences observed in Strain A replicates and complete absence of cases in Strain B and Strain C.

Model-based predictions of morbidity probability are illustrated in Fig 4C. Predicted probabilities remained close to zero across all ages and strains, reflecting the very low incidence of morbidity observed in the dataset.

Due to the extremely low number of morbidity events and the absence of cases in two strains, logistic regression analysis resulted in quasi-complete separation, yielding unstable parameter estimates. Therefore, morbidity outcomes were interpreted descriptively rather than inferentially.

### Temperature measurements

The relatively stable body temperatures observed across strains in Table 3 suggest that birds maintained physiological adaptation to the humid tropical environment.

## Discussion

### Growth performance of broiler strains

The significant difference observed in initial body weight among the broiler strains suggests genetic variation in early growth potential among commercial genotypes. Differences in chick weight at placement are common in commercial broiler strains and may reflect breeder flock age, egg size, or genetic growth characteristics [19,20]. In the present study, however, the initial differences did not translate into significant differences in final body weight or weight gain at market age. This indicates that all three strains were capable of achieving comparable growth performance when raised under similar management, nutrition, and environmental conditions.

Under humid-tropical production systems, environmental stressors such as high temperature and humidity can influence feed intake, metabolic activity, and overall growth performance of broiler chickens. Previous studies have shown that heat stress reduces feed intake and growth rate, thereby limiting the expression of genetic growth potential in broilers raised in tropical climates [21–24]. The absence of significant differences in growth performance among the strains in this study therefore suggests that environmental conditions may have moderated genetic differences in growth potential.

Feed conversion ratio did not differ significantly among strains, although Cobb 500 and Ross 308 showed numerically better efficiency compared with Hubbard. Modern commercial broiler strains have undergone extensive genetic selection for feed efficiency and rapid growth, resulting in relatively small differences in feed utilization under comparable management conditions [25,26]. These findings indicate that all three broiler strains evaluated in this study are capable of maintaining acceptable growth performance under humid-tropical production conditions, provided that adequate nutrition and management practices are implemented.

### Growth modeling

The Gompertz growth model provided a useful description of the growth trajectories of the broiler strains. Differences in asymptotic weight and inflection points among the strains reflect variations in growth dynamics and tissue deposition rates. Such differences are commonly observed among commercial broiler genotypes due to genetic selection for growth rate, muscle development, and feed efficiency [27,28].

In tropical production systems, however, environmental factors can influence the extent to which genetic growth potential is expressed. High ambient temperature and humidity may increase maintenance energy requirements and reduce feed intake, leading to lower asymptotic body weights compared with those reported under temperate conditions [8]. The slightly lower asymptotic weights observed in this study therefore likely reflect the influence of humid-tropical environmental conditions on broiler growth.

Despite these environmental constraints, the overall growth patterns observed among the strains were consistent with typical broiler growth curves reported in commercial production systems. This suggests that modern broiler strains maintain relatively stable growth dynamics even under challenging climatic conditions, although environmental stress may reduce the magnitude of growth.

### Carcass characteristics

The carcass evaluation revealed no significant differences among the three broiler strains in dressed weight, dressing percentage, or major carcass components, indicating comparable carcass yield under identical management conditions. This supports the premise that modern commercial broiler strains have been intensively selected for uniform growth and carcass traits, resulting in minimal variation among genotypes [29,30].

In tropical production systems, carcass performance is strongly influenced by environmental conditions, particularly heat stress, which can reduce feed intake and growth rate [31,32]. However, the absence of significant strain differences in the present study suggests that with adequate nutrition and proper management, these commercial strains can achieve similar carcass yields even under humid-tropical conditions.

Furthermore, the strong positive correlations observed among carcass weight traits indicate that growth performance remains a key determinant of carcass yield across strains. This reinforces the practical implication that improving overall body weight through better feeding and management will directly enhance carcass output, regardless of genotype [33–37].

Collectively, these findings suggest that poultry farmers in tropical environments can prioritize management practices over strain selection when aiming to optimize carcass yield, as Hubbard, Cobb 500, and Ross 308 strains demonstrate comparable performance under appropriate production conditions.

### Internal organ development

Significant differences in intestinal and liver weights were observed among the strains, suggesting variation in digestive and metabolic capacity. The digestive system plays a critical role in nutrient utilization and growth efficiency in broiler chickens. Larger intestinal development may enhance nutrient absorption capacity, thereby supporting growth and metabolic activity [38].

The relatively higher intestinal and liver weights observed in Ross 308 may indicate greater digestive capacity or metabolic activity, which could contribute to improved adaptation under humid-tropical conditions. Environmental factors such as heat stress may also influence organ development, as birds may exhibit physiological adjustments to maintain nutrient utilization and metabolic balance under stressful conditions [39,40].

Despite these differences, the weights of other internal organs such as the heart, gizzard, and spleen did not differ significantly among strains. This suggests that essential physiological functions related to circulation, digestion, and immune response were generally similar across the three broiler genotypes.

### Correlations among traits

The correlation analysis demonstrated strong positive relationships between body weight, breast weight, and dressing percentage. This indicates that heavier birds tend to produce higher quantities of marketable meat, which is consistent with the genetic selection goals of modern broiler breeding programs [41]. These relationships highlight the importance of body weight as a key determinant of carcass yield and economic value in broiler production.

The negative correlation observed between body weight and feed conversion ratio further indicates that birds with higher body weights tended to utilize feed more efficiently. Improved feed efficiency is a major objective of modern broiler breeding programs and contributes significantly to reducing production costs [42].

Additionally, positive correlations between liver weight and growth traits suggest increased metabolic activity in faster-growing birds, reflecting the role of the liver in nutrient metabolism and energy regulation.

### Mortality performance

Mortality varied among broiler strains under tropical production conditions, with Hubbard recording the highest mortality, followed by Cobb 500, while Ross 308 showed the lowest mortality. Although these differences were not statistically significant, the observed trends suggest variation in strain adaptability under humid tropical environments [10,24,29].

The higher mortality observed in Hubbard, particularly at early growth stages, may reflect greater sensitivity to environmental stress typical of tropical production systems, while the consistently lower mortality in Ross 308 suggests improved resilience [13,14].

These findings indicate that broiler production in tropical regions should prioritize strains with better survivability under heat stress, alongside appropriate management practices such as effective brooding temperature control, adequate ventilation, and optimized stocking density.

Overall, the results highlight that although growth performance may be similar across strains, differences in survivability are important considerations for optimizing broiler production in tropical environments.

### Morbidity performance

Morbidity incidence was minimal across all broiler strains, with only a few isolated cases observed in Hubbard and none in Cobb 500 and Ross 308. This generally reflects good flock health and effective management practices under the study conditions.

The absence of morbidity in Cobb 500 and Ross 308 may suggest better adaptability to the tropical production environment [14], although the low overall incidence limits definitive conclusions.

These findings emphasize the importance of biosecurity and environmental management in minimizing disease occurrence in broiler production systems.

### Physiological responses and heat stress adaptation

Body temperature measurements provide valuable insight into the thermoregulatory capacity of broiler chickens under hot and humid environmental conditions. In tropical regions, ambient temperatures frequently exceed the thermoneutral zone of broilers, resulting in increased heat stress and metabolic strain [43].

Maintaining stable body temperature is essential for sustaining growth performance and physiological homeostasis. Variations in body temperature among the strains may reflect differences in metabolic heat production, thermoregulation efficiency, and adaptability to heat stress. Broiler strains that maintain relatively stable body temperatures under high environmental temperatures may exhibit improved tolerance to tropical climatic conditions.

These findings highlight the importance of environmental management strategies such as improved ventilation, stocking density control, and nutritional adjustments to mitigate heat stress and maintain broiler productivity in humid-tropical production systems.

## Conclusions

This study demonstrated that while growth performance, feed efficiency, and carcass traits did not differ significantly among broiler strains under humid tropical conditions, important biological differences were evident. Cobb 500 (Strain B) showed superior growth potential, as reflected in higher numerical weight gain and final body weight, which was further supported by the Gompertz model indicating a higher asymptotic weight, faster growth rate, and earlier inflection point.

In contrast, health outcomes favored Ross 308 (Strain C), which recorded the lowest mortality and no morbidity, while Hubbard (Strain A) exhibited the highest mortality and the only cases of morbidity. Although these differences were not statistically significant, they indicate variation in adaptability and resilience among strains under tropical conditions.

Overall, the findings suggest that broiler strain selection in tropical production systems should balance growth performance with survivability and environmental adaptability. While Cobb 500 may offer advantages in growth, strains such as Ross 308 may provide improved resilience and reduced mortality risk. Therefore, optimizing broiler production in tropical environments requires both appropriate strain selection and effective management practices, including ventilation, brooding control, and nutrition, to enhance productivity and minimize losses.

## Supporting information

S1 Dataset Raw experimental data underlying the findings of this study, including growth performance, economic efficiency, carcass characteristics, internal organ measurements, and mortality and morbidity records for the three broiler strains. Data are provided in separate worksheets within the Excel workbook. (XLSX)
