# Supplementary material for: Integrated assessment of growth performance, carcass characteristics, and economic efficiency of three broiler strains raised under humid-tropical conditions
Source: PLoS One. 2026 Jun 22;21(6):e0351927. doi: 10.1371/journal.pone.0351927 (PMC13286170; doi:10.1371/journal.pone.0351927)
Supplement: Attachment — Submitted filename: Response to Reviewers.docx [file pone.0351927.s002.docx]

**Response to Editor and Reviewers**

**Manuscript ID:** PONE-D-26-01344R1
**Title:** Integrated Assessment of Growth Performance, Carcass Characteristics, and Economic Efficiency of Three Broiler Strains Raised Under Humid-Tropical Conditions

We sincerely thank the academic editor and both reviewers for their thorough evaluation and constructive comments. We have carefully revised the manuscript to address all concerns raised. All changes have been incorporated in the revised manuscript and highlighted in the tracked version.

Below, we provide a detailed, point-by-point response.

**Response to Reviewer 1**

**Comment 1**

In the introduction and discussion, describe more about the tropical conditions and the differences between commercial farms in Ghana.

**Response**

Thank you for this helpful suggestion. Additional information describing the humid-tropical production environment and common management systems used in Ghanaian poultry farms has been added to the Introduction to provide clearer context for the study.

These additions appear in the Introduction section (Lines 52-105).

**Comment 2**

Lines 54-56: Citation is missing. Add suggested references.

**Response**

The suggested references have been incorporated into the Introduction to strengthen the background and provide updated scientific support.

The new citations have been inserted in the Introduction (Lines 62-67).

**Comment 3**

The ethics statement is general and does not provide a specific permission number.

**Response**

This has been corrected. A specific ethical approval number has now been included: Approval No: KNUST-0018.

This clarification has been added to the Ethics Statement within the Methods section (Lines 108-111).

**Comment 4**

Only 5 birds per strain were used for carcass analysis, which is insufficient.

**Response**

We appreciate this observation. The selected birds were representative of the average body weight of each treatment group to minimize sampling bias. Similar sample sizes have been used in previous broiler carcass evaluation studies where birds are selected based on average live weight to represent the experimental population.

This explanation has been added in the Carcass and internal organ traits measurements subsection of the Materials and Methods section (Lines 182-189).

**Comment 5**

The results or discussion should better explain differences in growth performance among strains.

**Response**

This has now been addressed in the Discussion. We clarified that:

- Initial weight differences existed
- Environmental conditions (heat stress) likely moderated genetic potential

Thus, lack of significant differences is biologically explained.

These additions appear in the Discussion subsection “Growth Performance of Broiler Strains” (Lines 504-525).

**Comment 6**

Why are there different values between the final body weight in Table 5 and the live weight in Table 6?

**Response**

We thank the reviewer for this observation. To eliminate potential confusion and improve clarity, the parameter previously referred to as “live weight” in Table 6 has been removed from the revised manuscript.

The revised Table 6 now presents only post-slaughter carcass measurements (e.g., dressed weight, de-feathered weight, and carcass parts), which are directly relevant to carcass evaluation.

This revision ensures clearer distinction between Growth performance data (Table 5; based on all birds), and Carcass trait data (Table 6; based on sampled birds) and eliminates any ambiguity arising from differences in measurement basis.

The relevant values are presented in Table 5 (final body weight) and Table 6 (pre-slaughter live weight) of the Results section (Lines 341-356).

**Comment 7**

Divide discussion into subchapters.

**Response**

The Discussion has been fully restructured into clear subsections:

- Growth Performance of Broiler Strains
- Growth Modeling
- Carcass Characteristics
- Internal Organ Development
- Correlations among Traits
- Mortality Performance
- Morbidity Performance
- Physiological Responses

These structural revisions are reflected throughout the Discussion section (Lines 503-623).

**Comment 8**

Conclusion does not clearly state best strain.

**Response**

The Conclusion has been revised to provide a balanced interpretation:

- Cobb 500 → better growth potential
- Ross 308 → better survivability and adaptability

We intentionally avoided a single “best” strain because performance depends on production priorities (growth vs survival).

These revisions appear in the Conclusion section (Lines 625-640).

**Response to Reviewer 2**

**Major Comments**

1. **Experimental unit clarification**

**Response**

We thank the reviewer for this important clarification. The manuscript has been revised to explicitly define the experimental and observational units.

For growth performance variables (body weight, feed intake, and feed conversion ratio), the pen (replicate) was used as the experimental unit, as birds were group-housed and managed collectively within each pen. Accordingly, statistical analyses for growth traits were conducted using pen-level data (i.e., one observation per replicate per strain), ensuring independence of experimental units.

For carcass and internal organ measurements, individual birds were treated as observational units, as these measurements were obtained from randomly selected birds at slaughter.

Regarding sex distribution, birds were not sex-separated during the experimental period in order to simulate practical commercial production systems. However, for carcass evaluation, both male and female birds were randomly selected from each strain to ensure representative sampling.

These clarifications have been incorporated into the Experimental Design section of the revised manuscript (Lines 140-150).

**2. Initial body weight differences**

Initial Body Weight differences

**Response**

We appreciate the suggestion regarding the inclusion of initial body weight as a covariate. We used the initial body weight as a covariate for the analysis.

This clarification has been added to the Methods section in the Statistical Analysis subsection (Lines 265-305).

**3. Small carcass sample size**

Sample size for carcass and organ evaluation

**Response**

The selected birds were representative of the average body weight of each treatment group to minimize sampling bias. Similar sample sizes have been used in previous broiler carcass evaluation studies where birds are selected based on average live weight to represent the experimental population.

- Monteiro FA, Oliveira CH, Farias MRS, Fonseca L, Ramos BT, Braga GR, et al. Updated nutritional program improves performance, carcass yield, and profitability in male and female broilers. Poult Sci. 2026;105(2):106283. doi:10.1016/j.psj.2025.106283
- Grigore D, Mironeasa S, Ciurescu G, Ungureanu-Iuga M, Batariuc A, Babeanu NE. Carcass yield and meat quality of broiler chicks supplemented with yeasts bioproducts. Appl Sci. 2023;13(3):1607. doi:10.3390/app13031607

This explanation has been added in the Carcass and internal organ traits measurements subsection of the Materials and Methods section (Lines 182-189).

**4. Growth curve modeling clarification**

Growth modeling clarification

**Response**

The description of the Gompertz growth model has been clarified in the Analytical framework section, and the fitting procedure for each strain has been described more explicitly.

- Models fitted per strain using pooled data
- Added goodness-of-fit metrics: R² (pseudo), RMSE, AIC
- Included 95% confidence intervals
- Explained modeling approach clearly

These revisions appear in the Growth curve modeling subsection of the Materials and Methods (Lines 222-246).

**Comment 5**

The statistical model used for mortality analysis should be clearly described. Mortality data appear to be percentages but were analyzed using transformations. Clarify procedure and discuss causes of mortality.

**Response**

Thank you for this important observation. The mortality and morbidity analyses have been completely revised to improve statistical rigor and clarity.

- Statistical model clarification:

Mortality data were reanalyzed using a generalized linear model with a negative binomial distribution, which is more appropriate for count data and accounts for overdispersion. The model included strain, age, and their interaction as fixed effects. Results are now presented as incidence rate ratios (IRR) with 95% confidence intervals.

Morbidity data, recorded as binary outcomes, were analyzed using binomial logistic regression with strain, age, and their interaction as predictors.

- Correction of previous approach:

The earlier analysis based on percentage transformation has been removed. The revised approach ensures that the statistical methods align with the nature of the data (count and binary).

- Improved presentation:

Mortality and morbidity results are now presented using both descriptive summaries (totals, prevalence) and model-based inference, improving interpretability.

- Biological interpretation added:

Additional discussion has been included to explain that mortality occurred predominantly during the early brooding phase, likely due to factors such as brooding temperature fluctuations, chick quality, and early-life management stress, which are common challenges under humid tropical conditions.

These revisions have substantially improved the robustness and clarity of the mortality and morbidity analysis.

This clarification has been incorporated into the Materials and methods (Mortality and morbidity records; Lines 198-220), Results (Lines 429-497), and Discussion (Lines 589-610) sections discussing mortality and morbidity.

**6. Physiological temperature discussion**

**Response**

We thank the reviewer for this important observation. The discussion has been expanded to better interpret the physiological temperature measurements in relation to heat stress adaptation among broiler strains under humid-tropical conditions.

Additional explanations have been included to describe how body temperature reflects thermoregulation capacity and strain adaptability to high ambient temperatures typical of tropical production systems.

These revisions appear in the Discussion subsection “Physiological responses and heat stress adaptation” (Lines 611-623).

**7. Discussion depth and repetition**

**Response**

The discussion section has been carefully revised to reduce repetition and improve clarity. Greater emphasis has been placed on interpreting the results within the context of humid-tropical poultry production systems rather than relying primarily on comparisons with temperate environments.

Additionally, the practical implications of the findings for broiler farmers operating under tropical conditions have been highlighted, particularly regarding strain adaptability, survival, and production efficiency.

These revisions appear throughout the Discussion section (Lines 503-623).

**Minor Comments**

- **Language and grammar**

**Response**

The manuscript has been carefully edited for clarity, grammar, and scientific tone.

- **Formatting issues (decimals, units)**

**Response**

Corrected throughout: Decimal format standardized (e.g., 41.15), and Units standardized (kg, g, %, °C)

- **The morbidity table (Table 11) appears to contain an error: the parameter heading states “Mortality (%)” instead of “Morbidity (%)”.**

**Response**

Thank you for identifying this error. Following the reanalysis of the morbidity data using an appropriate binomial logistic regression approach, Table 11 (Line 489) has been fully updated to reflect the revised results. The corrected table now accurately presents morbidity outcomes and is consistent with the statistical methods described in the revised Methods section (Lines 209-220).

- **In the feed composition section, it would be useful to provide metabolizable energy for all diet phases and clearer feed formulation details.**

**Response**

Thank you for this helpful suggestion. The feed composition section has been revised and expanded to improve clarity and reproducibility.

Specifically:

Metabolizable energy (ME) values for all dietary phases (starter, grower, and finisher) have now been included (Line 172).

The ingredient composition and proportions of each diet have been clearly presented.

Additional details on feed formulation, including the inclusion of the toxin binder, have been clarified to ensure transparency.

These revisions provide a more comprehensive description of the diets used and enhance the reproducibility of the study.

- **References formatting**

**Response**

All references have been revised to match PLOS ONE style (Lines 654-774).

- **Figures and captions**

**Response**

All figures: Properly labeled, Referenced in text, and Captions improved for clarity

**EDITORIAL REQUIREMENTS**

**Funding statement correction**

**Response**

Funding-related text has been removed from Acknowledgments.

Updated statement (to include in cover letter):

“The authors received no specific funding for this work.”

**Data availability**

**Response**

Confirmed:

- All raw data supporting results are included as Supporting Information

Statement updated accordingly.

We believe that the manuscript has been substantially improved in response to all reviewers and academic editor comments. We respectfully submit the revised manuscript for reconsideration and hope it is now suitable for publication in PLOS ONE.

Sincerely,
Dennis Owusu Acheampong

(on behalf of all authors)
