# Supplementary material for: Integrated assessment of growth performance, carcass characteristics, and economic efficiency of three broiler strains raised under humid-tropical conditions
Source: PLoS One. 2026 Jun 22;21(6):e0351927. doi: 10.1371/journal.pone.0351927 (PMC13286170; doi:10.1371/journal.pone.0351927)
Supplement: Attachment — Submitted filename: Final Corrections.docx [file pone.0351927.s003.docx]

I observed that the authors did not include the markings in the manuscript indicating where the corrections requested in the previous review were made. Therefore, it was not possible to clearly identify the modifications performed. Thus, I am returning the manuscript so that the authors can resubmit the files with the corrections properly indicated in the document.

Sincerely

(MKS)
