# Supplementary material for: Integrated assessment of growth performance, carcass characteristics, and economic efficiency of three broiler strains raised under humid-tropical conditions
Source: PLoS One. 2026 Jun 22;21(6):e0351927. doi: 10.1371/journal.pone.0351927 (PMC13286170; doi:10.1371/journal.pone.0351927)
Supplement: Attachment — Submitted filename: Response to Reviewers.doc [file pone.0351927.s005.doc]

I have carefully examined the revised manuscript and the authors' responses to the reviewers' comments. The authors have adequately addressed all the concerns raised during the previous review process, and the revisions have improved the quality and clarity of the manuscript. In my opinion, all comments have been satisfactorily answered, and I have no further concerns regarding the manuscript. Therefore, I recommend its acceptance for publication.

Sincerely,

(MKS)
