# Supplementary material for: Integrated assessment of growth performance, carcass characteristics, and economic efficiency of three broiler strains raised under humid-tropical conditions
Source: PLoS One. 2026 Jun 22;21(6):e0351927. doi: 10.1371/journal.pone.0351927 (PMC13286170; doi:10.1371/journal.pone.0351927)
Supplement: Peer review history

# Peer review: Decision Letter 0

**DOI:** 10.1371/journal.pone.0351927.r001

**Authors:** Lamiaa Mostafa Radwan

23 Mar 2026

PONE-D-26-01344

Integrated assessment of growth performance, carcass characteristics and economic efficiency of three broiler breeds raised under humid-tropical conditions

PLOS One

Dear Dr. DENNIS OWUSU ACHEAMPONG, MPHIL.,

Thank you for submitting your manuscript to PLOS ONE. After careful consideration, we feel that it has merit but does not fully meet PLOS ONE’s publication criteria as it currently stands. Therefore, we invite you to submit a revised version of the manuscript that addresses the points raised during the review process.

==============================

Dear Dr. ACHEAMPONG,

Thank you for submitting your manuscript to PLOS ONE. After careful consideration, we have decided that your manuscript needs Major Revision

Kind regards,

Prof. Lamiaa Mostafa Radwan, Ph.D.

Academic Editor

PLOS ONE

**Reviewer1**

The study provides an interesting comparison of three commercial broiler hybrids, Hubbard, Cobb 500, and Ross 308, specifically under the tropical conditions of Ghana. The manuscript is well-structured, methodologically sound, and the results are supported by statistical analysis. The added value of the study is that the authors evaluate not only growth but also carcass traits, internal organ development, economic efficiency, and health status (mortality/morbidity).

Specific comments:

In the introduction and discussion, describe more about the tropical conditions and the differences between commercial farms in Ghana.

Lines 54-56: The citation is missing after the sentence. I recommend adding citations: https://doi.org/10.3389/fvets.2025.1517142 and https://doi.org/10.1186/s13568-024-01777-2.

The ethics statement is general and does not provide a specific permission number for the study.

Methods: Only 5 birds per strain were used for carcass analysis, which is an insufficient number for statistical evaluation.

Results: The results or discussion do not explain why the final body weight of strain A was higher than the others.

Why are there different values between the final body weight in Table 5 and the live weight in Table 6?

I recommend dividing the discussion into individual subchapters based on the presented results to make it clearer.

In conclusion, it is not clear which broiler chicken hybrid is best suited to tropical conditions.

**Reviewer2**

Comments to the Author

General Assessment

The manuscript entitled “Integrated assessment of growth performance, carcass characteristics and economic efficiency of three broiler breeds raised under humid-tropical conditions” evaluates the performance of three commercial broiler strains (Hubbard, Cobb 500, and Ross 308) under humid tropical conditions in Ghana. The study integrates growth performance, carcass traits, physiological indicators, mortality, morbidity, and growth curve modeling using the Gompertz function. The topic is relevant to poultry production in tropical regions and contributes useful information regarding genotype performance and adaptability.

The manuscript is generally well structured and addresses an important issue for poultry production systems in tropical climates. However, several methodological clarifications, improvements in statistical description, language editing, and strengthening of the discussion are required before the manuscript can be considered for publication.

Major Comments

1. Experimental design clarification

Although the manuscript states that a completely randomized design (CRD) was used with 300 birds and 5 replicates per strain, further clarification is needed regarding:

Whether the pen or individual bird was used as the experimental unit for statistical analysis.

How sex distribution was handled across replicates, especially since carcass sampling included both males and females.

Clarifying the experimental unit is essential to ensure the validity of the statistical analysis.

2. Initial body weight differences

The study reports significant differences in initial body weight among strains. This could potentially influence later performance results. The authors should clarify:

Whether initial body weight was used as a covariate in the statistical analysis.

If not, discuss how these differences may have affected growth performance comparisons.

3. Sample size for carcass and organ evaluation

Only five birds per strain were used for carcass and organ measurements. This sample size appears relatively small compared with the total population (300 birds).

The authors should:

Provide justification for the sample size.

Discuss the potential limitations of small sampling on statistical power and reliability of carcass trait comparisons.

4. Growth curve modeling

The Gompertz growth model is appropriate; however, several aspects require clarification:

The manuscript does not specify whether separate models were fitted for each replicate or for pooled data per strain.

Model goodness-of-fit indicators (e.g., R², AIC, RMSE) are not reported.

Confidence intervals or statistical comparison of Gompertz parameters among strains should be considered.

Including model fit statistics would improve the reliability of the growth modeling results.

5. Mortality and morbidity analysis

The mortality results indicate significant strain differences, while morbidity differences were non-significant. However:

The statistical model used for mortality analysis should be clearly described.

Mortality data appear to be percentages but were analyzed using transformations. The authors should clarify the exact procedure used.

Since mortality occurred mostly during the brooding phase, the authors should provide a brief discussion of possible causes (e.g., brooding temperature fluctuations, chick quality, or management factors).

6. Physiological temperature measurements

Body temperature measurements were collected but not discussed sufficiently in the results or discussion sections. The authors should:

Explain how body temperature relates to heat stress adaptation among strains.

Integrate these findings into the discussion.

7. Discussion depth

The discussion is extensive but occasionally repetitive and sometimes relies heavily on comparisons with temperate studies.

The authors should:

Focus more specifically on tropical production systems.

Avoid repeating similar interpretations across sections.

Highlight practical implications for poultry farmers in tropical regions.

Minor Comments

Several grammatical and stylistic issues are present throughout the manuscript. Professional language editing is recommended.

Some formatting inconsistencies exist, such as:

commas used instead of decimals (e.g., “41,15” instead of “41.15”)

inconsistent unit formatting (kg, g, %, ℃).

The morbidity table (Table 11) appears to contain an error: the parameter heading states “Mortality (%)” instead of “Morbidity (%)”.

In the feed composition section, it would be useful to provide:

metabolizable energy for all diet phases

clearer feed formulation details.

Some references appear incomplete or inconsistently formatted and should be checked according to the journal style.

The authors should verify all figure captions and ensure that each figure is clearly referenced in the text.

Overall Recommendation to Authors

The manuscript addresses an important topic and presents potentially valuable findings for broiler production in humid tropical environments. However, revisions are necessary to clarify the experimental design, strengthen the statistical analysis, and improve presentation and interpretation of results.

==============================

Please submit your revised manuscript by 23 April 2024. If you will need more time than this to complete your revisions, please reply to this message or contact the journal office at plosone@plos.org. When you're ready to submit your revision, log on to https://www.editorialmanager.com/pone/ and select the 'Submissions Needing Revision' folder to locate your manuscript file.

Please include the following items when submitting your revised manuscript:

- A letter that responds to each point raised by the academic editor and reviewer(s). You should upload this letter as a separate file labeled 'Response to Reviewers'.
- A marked-up copy of your manuscript that highlights changes made to the original version. You should upload this as a separate file labeled 'Revised Manuscript with Track Changes'.
- An unmarked version of your revised paper without tracked changes. You should upload this as a separate file labeled 'Manuscript'.

If you would like to make changes to your financial disclosure, please include your updated statement in your cover letter. Guidelines for resubmitting your figure files are available below the reviewer comments at the end of this letter.

If applicable, we recommend that you deposit your laboratory protocols in protocols.io to enhance the reproducibility of your results. Protocols.io assigns your protocol its own identifier (DOI) so that it can be cited independently in the future. For instructions see: https://journals.plos.org/plosone/s/submission-guidelines#loc-laboratory-protocols. Additionally, PLOS ONE offers an option for publishing peer-reviewed Lab Protocol articles, which describe protocols hosted on protocols.io. Read more information on sharing protocols at https://plos.org/protocols?utm_medium=editorial-email&utm_source=authorletters&utm_campaign=protocols.

We look forward to receiving your revised manuscript.

Kind regards,

Lamiaa Mostafa Radwan, Ph.D.

Academic Editor

PLOS One

Journal Requirements:

When submitting your revision, we need you to address these additional requirements.

1. Please ensure that your manuscript meets PLOS ONE's style requirements, including those for file naming. The PLOS ONE style templates can be found at

https://journals.plos.org/plosone/s/file?id=wjVg/PLOSOne_formatting_sample_main_body.pdf and

https://journals.plos.org/plosone/s/file?id=ba62/PLOSOne_formatting_sample_title_authors_affiliations.pdf

2. Thank you for stating the following in the Acknowledgments Section of your manuscript:

"We wish to thank Professor Rexford Assasie Oppong for supporting with the funding. Appreciation is also extended to the technical staff and farm attendants at the Department of Animal Science, Kwame Nkrumah University of Science and Technology (KNUST), for their support during on field study. 6"

We note that you have provided additional information within the Acknowledgements Section that is not currently declared in your Funding Statement. Please note that funding information should not appear in the Acknowledgments section or other areas of your manuscript. We will only publish funding information present in the Funding Statement section of the online submission form.

Please remove any funding-related text from the manuscript and let us know how you would like to update your Funding Statement. Currently, your Funding Statement reads as follows:

"The author(s) received no specific funding for this work."

Please include your amended statements within your cover letter; we will change the online submission form on your behalf.

3. Thank you for stating the following financial disclosure:

"The author(s) received no specific funding for this work."

At this time, please address the following queries:

a)        Please clarify the sources of funding (financial or material support) for your study. List the grants or organizations that supported your study, including funding received from your institution.

b)        State what role the funders took in the study. If the funders had no role in your study, please state: “The funders had no role in study design, data collection and analysis, decision to publish, or preparation of the manuscript.”

c)        If any authors received a salary from any of your funders, please state which authors and which funders.

d)        If you did not receive any funding for this study, please state: “The authors received no specific funding for this work.”

Please include your amended statements within your cover letter; we will change the online submission form on your behalf.

4. We note that your Data Availability Statement is currently as follows: "All relevant data are within the manuscript and its Supporting Information files."

Please confirm at this time whether or not your submission contains all raw data required to replicate the results of your study. Authors must share the “minimal data set” for their submission. PLOS defines the minimal data set to consist of the data required to replicate all study findings reported in the article, as well as related metadata and methods (https://journals.plos.org/plosone/s/data-availability#loc-minimal-data-set-definition).

For example, authors should submit the following data:

- The values behind the means, standard deviations and other measures reported;

- The values used to build graphs;

- The points extracted from images for analysis.

Authors do not need to submit their entire data set if only a portion of the data was used in the reported study.

If your submission does not contain these data, please either upload them as Supporting Information files or deposit them to a stable, public repository and provide us with the relevant URLs, DOIs, or accession numbers. For a list of recommended repositories, please see https://journals.plos.org/plosone/s/recommended-repositories.

If there are ethical or legal restrictions on sharing a de-identified data set, please explain them in detail (e.g., data contain potentially sensitive information, data are owned by a third-party organization, etc.) and who has imposed them (e.g., an ethics committee). Please also provide contact information for a data access committee, ethics committee, or other institutional body to which data requests may be sent. If data are owned by a third party, please indicate how others may request data access.

5. If the reviewer comments include a recommendation to cite specific previously published works, please review and evaluate these publications to determine whether they are relevant and should be cited. There is no requirement to cite these works unless the editor has indicated otherwise.

Additional Editor Comments :

Dear Dr., DENNIS OWUSU ACHEAMPONG, MPHIL.

Thank you for submitting your manuscript to PLOS ONE. After careful consideration, we have decided that your manuscript needs Major Revision.

Kind regards,

Prof. Lamiaa Mostafa Radwan, Ph.D.

Academic Editor

PLOS ONE

Reviewer1

The study provides an interesting comparison of three commercial broiler hybrids, Hubbard, Cobb 500, and Ross 308, specifically under the tropical conditions of Ghana. The manuscript is well-structured, methodologically sound, and the results are supported by statistical analysis. The added value of the study is that the authors evaluate not only growth but also carcass traits, internal organ development, economic efficiency, and health status (mortality/morbidity).

Specific comments:

In the introduction and discussion, describe more about the tropical conditions and the differences between commercial farms in Ghana.

Lines 54-56: The citation is missing after the sentence. I recommend adding citations: https://doi.org/10.3389/fvets.2025.1517142 and https://doi.org/10.1186/s13568-024-01777-2.

The ethics statement is general and does not provide a specific permission number for the study.

Methods: Only 5 birds per strain were used for carcass analysis, which is an insufficient number for statistical evaluation.

Results: The results or discussion do not explain why the final body weight of strain A was higher than the others.

Why are there different values between the final body weight in Table 5 and the live weight in Table 6?

I recommend dividing the discussion into individual subchapters based on the presented results to make it clearer.

In conclusion, it is not clear which broiler chicken hybrid is best suited to tropical conditions.

Reviewer2

Comments to the Author

General Assessment

The manuscript entitled “Integrated assessment of growth performance, carcass characteristics and economic efficiency of three broiler breeds raised under humid-tropical conditions” evaluates the performance of three commercial broiler strains (Hubbard, Cobb 500, and Ross 308) under humid tropical conditions in Ghana. The study integrates growth performance, carcass traits, physiological indicators, mortality, morbidity, and growth curve modeling using the Gompertz function. The topic is relevant to poultry production in tropical regions and contributes useful information regarding genotype performance and adaptability.

The manuscript is generally well structured and addresses an important issue for poultry production systems in tropical climates. However, several methodological clarifications, improvements in statistical description, language editing, and strengthening of the discussion are required before the manuscript can be considered for publication.

Major Comments

1. Experimental design clarification

Although the manuscript states that a completely randomized design (CRD) was used with 300 birds and 5 replicates per strain, further clarification is needed regarding:

Whether the pen or individual bird was used as the experimental unit for statistical analysis.

How sex distribution was handled across replicates, especially since carcass sampling included both males and females.

Clarifying the experimental unit is essential to ensure the validity of the statistical analysis.

2. Initial body weight differences

The study reports significant differences in initial body weight among strains. This could potentially influence later performance results. The authors should clarify:

Whether initial body weight was used as a covariate in the statistical analysis.

If not, discuss how these differences may have affected growth performance comparisons.

3. Sample size for carcass and organ evaluation

Only five birds per strain were used for carcass and organ measurements. This sample size appears relatively small compared with the total population (300 birds).

The authors should:

Provide justification for the sample size.

Discuss the potential limitations of small sampling on statistical power and reliability of carcass trait comparisons.

4. Growth curve modeling

The Gompertz growth model is appropriate; however, several aspects require clarification:

The manuscript does not specify whether separate models were fitted for each replicate or for pooled data per strain.

Model goodness-of-fit indicators (e.g., R², AIC, RMSE) are not reported.

Confidence intervals or statistical comparison of Gompertz parameters among strains should be considered.

Including model fit statistics would improve the reliability of the growth modeling results.

5. Mortality and morbidity analysis

The mortality results indicate significant strain differences, while morbidity differences were non-significant. However:

The statistical model used for mortality analysis should be clearly described.

Mortality data appear to be percentages but were analyzed using transformations. The authors should clarify the exact procedure used.

Since mortality occurred mostly during the brooding phase, the authors should provide a brief discussion of possible causes (e.g., brooding temperature fluctuations, chick quality, or management factors).

6. Physiological temperature measurements

Body temperature measurements were collected but not discussed sufficiently in the results or discussion sections. The authors should:

Explain how body temperature relates to heat stress adaptation among strains.

Integrate these findings into the discussion.

7. Discussion depth

The discussion is extensive but occasionally repetitive and sometimes relies heavily on comparisons with temperate studies.

The authors should:

Focus more specifically on tropical production systems.

Avoid repeating similar interpretations across sections.

Highlight practical implications for poultry farmers in tropical regions.

Minor Comments

Several grammatical and stylistic issues are present throughout the manuscript. Professional language editing is recommended.

Some formatting inconsistencies exist, such as:

commas used instead of decimals (e.g., “41,15” instead of “41.15”)

inconsistent unit formatting (kg, g, %, ℃).

The morbidity table (Table 11) appears to contain an error: the parameter heading states “Mortality (%)” instead of “Morbidity (%)”.

In the feed composition section, it would be useful to provide:

metabolizable energy for all diet phases

clearer feed formulation details.

Some references appear incomplete or inconsistently formatted and should be checked according to the journal style.

The authors should verify all figure captions and ensure that each figure is clearly referenced in the text.

Overall Recommendation to Authors

The manuscript addresses an important topic and presents potentially valuable findings for broiler production in humid tropical environments. However, revisions are necessary to clarify the experimental design, strengthen the statistical analysis, and improve presentation and interpretation of results.

[Note: HTML markup is below. Please do not edit.]

Reviewers' comments:

Reviewer's Responses to Questions

**Comments to the Author**

1. Is the manuscript technically sound, and do the data support the conclusions?

The manuscript must describe a technically sound piece of scientific research with data that supports the conclusions. Experiments must have been conducted rigorously, with appropriate controls, replication, and sample sizes. The conclusions must be drawn appropriately based on the data presented.

Reviewer #1: Yes

Reviewer #2: Partly

**********

2. Has the statistical analysis been performed appropriately and rigorously?

Reviewer #1: Yes

Reviewer #2: No

**********

3. Have the authors made all data underlying the findings in their manuscript fully available?

The PLOS Data policy requires authors to make all data underlying the findings described in their manuscript fully available without restriction, with rare exception (please refer to the Data Availability Statement in the manuscript PDF file). The data should be provided as part of the manuscript or its supporting information, or deposited to a public repository. For example, in addition to summary statistics, the data points behind means, medians and variance measures should be available. If there are restrictions on publicly sharing data—e.g. participant privacy or use of data from a third party—those must be specified.

Reviewer #1: Yes

Reviewer #2: Yes

**********

4. Is the manuscript presented in an intelligible fashion and written in standard English?

PLOS ONE does not copyedit accepted manuscripts, so the language in submitted articles must be clear, correct, and unambiguous. Any typographical or grammatical errors should be corrected at revision, so please note any specific errors here.

Reviewer #1: Yes

Reviewer #2: No

**********

5. Review Comments to the Author

Please use the space provided to explain your answers to the questions above. You may also include additional comments for the author, including concerns about dual publication, research ethics, or publication ethics. (Please upload your review as an attachment if it exceeds 20,000 characters)

Reviewer #1: The study provides an interesting comparison of three commercial broiler hybrids, Hubbard, Cobb 500, and Ross 308, specifically under the tropical conditions of Ghana. The manuscript is well-structured, methodologically sound, and the results are supported by statistical analysis. The added value of the study is that the authors evaluate not only growth but also carcass traits, internal organ development, economic efficiency, and health status (mortality/morbidity).

Specific comments:

In the introduction and discussion, describe more about the tropical conditions and the differences between commercial farms in Ghana.

Lines 54-56: The citation is missing after the sentence. I recommend adding citations: https://doi.org/10.3389/fvets.2025.1517142 and https://doi.org/10.1186/s13568-024-01777-2.

The ethics statement is general and does not provide a specific permission number for the study.

Methods: Only 5 birds per strain were used for carcass analysis, which is an insufficient number for statistical evaluation.

Results: The results or discussion do not explain why the final body weight of strain A was higher than the others.

Why are there different values between the final body weight in Table 5 and the live weight in Table 6?

I recommend dividing the discussion into individual subchapters based on the presented results to make it clearer.

In conclusion, it is not clear which broiler chicken hybrid is best suited to tropical conditions.

Reviewer #2: Comments to the Author

General Assessment

The manuscript entitled “Integrated assessment of growth performance, carcass characteristics and economic efficiency of three broiler breeds raised under humid-tropical conditions” evaluates the performance of three commercial broiler strains (Hubbard, Cobb 500, and Ross 308) under humid tropical conditions in Ghana. The study integrates growth performance, carcass traits, physiological indicators, mortality, morbidity, and growth curve modeling using the Gompertz function. The topic is relevant to poultry production in tropical regions and contributes useful information regarding genotype performance and adaptability.

The manuscript is generally well structured and addresses an important issue for poultry production systems in tropical climates. However, several methodological clarifications, improvements in statistical description, language editing, and strengthening of the discussion are required before the manuscript can be considered for publication.

Major Comments

1. Experimental design clarification

Although the manuscript states that a completely randomized design (CRD) was used with 300 birds and 5 replicates per strain, further clarification is needed regarding:

Whether the pen or individual bird was used as the experimental unit for statistical analysis.

How sex distribution was handled across replicates, especially since carcass sampling included both males and females.

Clarifying the experimental unit is essential to ensure the validity of the statistical analysis.

2. Initial body weight differences

The study reports significant differences in initial body weight among strains. This could potentially influence later performance results. The authors should clarify:

Whether initial body weight was used as a covariate in the statistical analysis.

If not, discuss how these differences may have affected growth performance comparisons.

3. Sample size for carcass and organ evaluation

Only five birds per strain were used for carcass and organ measurements. This sample size appears relatively small compared with the total population (300 birds).

The authors should:

Provide justification for the sample size.

Discuss the potential limitations of small sampling on statistical power and reliability of carcass trait comparisons.

4. Growth curve modeling

The Gompertz growth model is appropriate; however, several aspects require clarification:

The manuscript does not specify whether separate models were fitted for each replicate or for pooled data per strain.

Model goodness-of-fit indicators (e.g., R², AIC, RMSE) are not reported.

Confidence intervals or statistical comparison of Gompertz parameters among strains should be considered.

Including model fit statistics would improve the reliability of the growth modeling results.

5. Mortality and morbidity analysis

The mortality results indicate significant strain differences, while morbidity differences were non-significant. However:

The statistical model used for mortality analysis should be clearly described.

Mortality data appear to be percentages but were analyzed using transformations. The authors should clarify the exact procedure used.

Since mortality occurred mostly during the brooding phase, the authors should provide a brief discussion of possible causes (e.g., brooding temperature fluctuations, chick quality, or management factors).

6. Physiological temperature measurements

Body temperature measurements were collected but not discussed sufficiently in the results or discussion sections. The authors should:

Explain how body temperature relates to heat stress adaptation among strains.

Integrate these findings into the discussion.

7. Discussion depth

The discussion is extensive but occasionally repetitive and sometimes relies heavily on comparisons with temperate studies.

The authors should:

Focus more specifically on tropical production systems.

Avoid repeating similar interpretations across sections.

Highlight practical implications for poultry farmers in tropical regions.

Minor Comments

Several grammatical and stylistic issues are present throughout the manuscript. Professional language editing is recommended.

Some formatting inconsistencies exist, such as:

commas used instead of decimals (e.g., “41,15” instead of “41.15”)

inconsistent unit formatting (kg, g, %, ℃).

The morbidity table (Table 11) appears to contain an error: the parameter heading states “Mortality (%)” instead of “Morbidity (%)”.

In the feed composition section, it would be useful to provide:

metabolizable energy for all diet phases

clearer feed formulation details.

Some references appear incomplete or inconsistently formatted and should be checked according to the journal style.

The authors should verify all figure captions and ensure that each figure is clearly referenced in the text.

Overall Recommendation to Authors

The manuscript addresses an important topic and presents potentially valuable findings for broiler production in humid tropical environments. However, revisions are necessary to clarify the experimental design, strengthen the statistical analysis, and improve presentation and interpretation of results.

**********

6. PLOS authors have the option to publish the peer review history of their article (what does this mean?). If published, this will include your full peer review and any attached files.

If you choose “no”, your identity will remain anonymous but your review may still be made public.

**Do you want your identity to be public for this peer review?**   For information about this choice, including consent withdrawal, please see our Privacy Policy.

Reviewer #1: No

Reviewer #2: **Yes:**   Dr. Manish K. Singh

**********

[NOTE: If reviewer comments were submitted as an attachment file, they will be attached to this email and accessible via the submission site. Please log into your account, locate the manuscript record, and check for the action link "View Attachments". If this link does not appear, there are no attachment files.]

To ensure your figures meet our technical requirements, please review our figure guidelines: https://journals.plos.org/plosone/s/figures

You may also use PLOS’s free figure tool, NAAS, to help you prepare publication quality figures: https://journals.plos.org/plosone/s/figures#loc-tools-for-figure-preparation.

NAAS will assess whether your figures meet our technical requirements by comparing each figure against our figure specifications.

# Author comment: Author response to Decision Letter 1

**DOI:** 10.1371/journal.pone.0351927.r002

4 May 2026

Response to Editor and Reviewers

Manuscript ID: PONE-D-26-01344R1

Title: Integrated Assessment of Growth Performance, Carcass Characteristics, and Economic Efficiency of Three Broiler Strains Raised Under Humid-Tropical Conditions

We sincerely thank the academic editor and both reviewers for their thorough evaluation and constructive comments. We have carefully revised the manuscript to address all concerns raised. All changes have been incorporated in the revised manuscript and highlighted in the tracked version.

Below, we provide a detailed, point-by-point response.

Response to Reviewer 1

Comment 1

In the introduction and discussion, describe more about the tropical conditions and the differences between commercial farms in Ghana.

Response

Thank you for this helpful suggestion. Additional information describing the humid-tropical production environment and common management systems used in Ghanaian poultry farms has been added to the Introduction to provide clearer context for the study.

These additions appear in the Introduction section (Lines 52-105).

Comment 2

Lines 54-56: Citation is missing. Add suggested references.

Response

The suggested references have been incorporated into the Introduction to strengthen the background and provide updated scientific support.

The new citations have been inserted in the Introduction (Lines 62-67).

Comment 3

The ethics statement is general and does not provide a specific permission number.

Response

This has been corrected. A specific ethical approval number has now been included: Approval No: KNUST-0018.

This clarification has been added to the Ethics Statement within the Methods section (Lines 108-111).

Comment 4

Only 5 birds per strain were used for carcass analysis, which is insufficient.

Response

We appreciate this observation. The selected birds were representative of the average body weight of each treatment group to minimize sampling bias. Similar sample sizes have been used in previous broiler carcass evaluation studies where birds are selected based on average live weight to represent the experimental population.

This explanation has been added in the Carcass and internal organ traits measurements subsection of the Materials and Methods section (Lines 181-188).

Comment 5

The results or discussion should better explain differences in growth performance among strains.

Response

This has now been addressed in the Discussion. We clarified that:

• Initial weight differences existed

• Environmental conditions (heat stress) likely moderated genetic potential

Thus, lack of significant differences is biologically explained.

These additions appear in the Discussion subsection “Growth Performance of Broiler Strains” (Lines 482-496).

Comment 6

Why are there different values between the final body weight in Table 5 and the live weight in Table 6?

Response

We thank the reviewer for this observation. To eliminate potential confusion and improve clarity, the parameter previously referred to as “live weight” in Table 6 has been removed from the revised manuscript.

The revised Table 6 now presents only post-slaughter carcass measurements (e.g., dressed weight, de-feathered weight, and carcass parts), which are directly relevant to carcass evaluation.

This revision ensures clearer distinction between Growth performance data (Table 5; based on all birds), and Carcass trait data (Table 6; based on sampled birds) and eliminates any ambiguity arising from differences in measurement basis.

The relevant values are presented in Table 5 (final body weight) and Table 6 (pre-slaughter live weight) of the Results section (Lines 326-341).

Comment 7

Divide discussion into subchapters.

Response

The Discussion has been fully restructured into clear subsections:

• Growth Performance of Broiler Strains

• Growth Modeling

• Carcass Characteristics

• Internal Organ Development

• Correlations among Traits

• Mortality Performance

• Morbidity Performance

• Physiological Responses

These structural revisions are reflected throughout the Discussion section (Lines 481-598).

Comment 8

Conclusion does not clearly state best strain.

Response

The Conclusion has been revised to provide a balanced interpretation:

• Cobb 500 → better growth potential

• Ross 308 → better survivability and adaptability

We intentionally avoided a single “best” strain because performance depends on production priorities (growth vs survival).

These revisions appear in the Conclusion section (Lines 600-615).

Response to Reviewer 2

Major Comments

1. Experimental unit clarification

Response

We thank the reviewer for this important clarification. The manuscript has been revised to explicitly define the experimental and observational units.

For growth performance variables (body weight, feed intake, and feed conversion ratio), the pen (replicate) was used as the experimental unit, as birds were group-housed and managed collectively within each pen. Accordingly, statistical analyses for growth traits were conducted using pen-level data (i.e., one observation per replicate per strain), ensuring independence of experimental units.

For carcass and internal organ measurements, individual birds were treated as observational units, as these measurements were obtained from randomly selected birds at slaughter.

Regarding sex distribution, birds were not sex-separated during the experimental period in order to simulate practical commercial production systems. However, for carcass evaluation, both male and female birds were randomly selected from each strain to ensure representative sampling.

These clarifications have been incorporated into the Experimental Design section of the revised manuscript (Lines 140-150).

2. Initial body weight differences

Initial Body Weight differences

Response

We appreciate the suggestion regarding the inclusion of initial body weight as a covariate. We used the initial body weight as a covariate for the analysis.

This clarification has been added to the Methods section in the Statistical Analysis subsection (Lines 253-293).

3. Small carcass sample size

Sample size for carcass and organ evaluation

Response

The selected birds were representative of the average body weight of each treatment group to minimize sampling bias. Similar sample sizes have been used in previous broiler carcass evaluation studies where birds are selected based on average live weight to represent the experimental population.

• Monteiro FA, Oliveira CH, Farias MRS, Fonseca L, Ramos BT, Braga GR, et al. Updated nutritional program improves performance, carcass yield, and profitability in male and female broilers. Poult Sci. 2026;105(2):106283. doi:10.1016/j.psj.2025.106283

• Grigore D, Mironeasa S, Ciurescu G, Ungureanu-Iuga M, Batariuc A, Babeanu NE. Carcass yield and meat quality of broiler chicks supplemented with yeasts bioproducts. Appl Sci. 2023;13(3):1607. doi:10.3390/app13031607

This explanation has been added in the Carcass and internal organ traits measurements subsection of the Materials and Methods section (Lines 181-188).

4. Growth curve modeling clarification

Growth modeling clarification

Response

The description of the Gompertz growth model has been clarified in the Analytical framework section, and the fitting procedure for each strain has been described more explicitly.

• Models fitted per strain using pooled data

• Added goodness-of-fit metrics: R² (pseudo), RMSE, AIC

• Included 95% confidence intervals

• Explained modeling approach clearly

These revisions appear in the Growth curve modeling subsection of the Materials and Methods (Lines 221-245).

Comment 5

The statistical model used for mortality analysis should be clearly described. Mortality data appear to be percentages but were analyzed using transformations. Clarify procedure and discuss causes of mortality.

Response

Thank you for this important observation. The mortality and morbidity analyses have been completely revised to improve statistical rigor and clarity.

• Statistical model clarification:

Mortality data were reanalyzed using a generalized linear model with a negative binomial distribution, which is more appropriate for count data and accounts for overdispersion. The model included strain, age, and their interaction as fixed effects. Results are now presented as incidence rate ratios (IRR) with 95% confidence intervals.

Morbidity data, recorded as binary outcomes, were analyzed using binomial logistic regression with strain, age, and their interaction as predictors.

• Correction of previous approach:

The earlier analysis based on percentage transformation has been removed. The revised approach ensures that the statistical methods align with the nature of the data (count and binary).

• Improved presentation:

Mortality and morbidity results are now presented using both descriptive summaries (totals, prevalence) and model-based inference, improving interpretability.

• Biological interpretation added:

Additional discussion has been included to explain that mortality occurred predominantly during the early brooding phase, likely due to factors such as brooding temperature fluctuations, chick quality, and early-life management stress, which are common challenges under humid tropical conditions.

These revisions have substantially improved the robustness and clarity of the mortality and morbidity analysis.

This clarification has been incorporated into the Materials and methods (Mortality and morbidity records; Lines 197-219), Results (Lines 411-476), and Discussion (Lines 564-585) sections discussing mortality and morbidity.

6. Physiological temperature discussion

Response

We thank the reviewer for this important observation. The discussion has been expanded to better interpret the physiological temperature measurements in relation to heat stress adaptation among broiler strains under humid-tropical conditions.

Additional explanations have been included to describe how body temperature reflects thermoregulation capacity and strain adaptability to high ambient temperatures typical of tropical production systems.

These revisions appear in the Discussion subsection “Physiological responses and heat stress adaptation” (Lines 586-598).

7. Discussion depth and repetition

Response

The discussion section has been carefully revised to reduce repetition and improve clarity. Greater emphasis has been placed on interpreting the results within the context of humid-tropical poultry production systems rather than relying primarily on comparisons with temperate environments.

Additionally, the practical implications of the findings for broiler farmers operating under tropical conditions have been highlighted, particularly regarding strain adaptability, survival, and production efficiency.

These revisions appear throughout the Discussion section (Lines 481-598).

Minor Comments

• Language and grammar

Response

The manuscript has been carefully edited for clarity, grammar, and scientific tone.

• Formatting issues (decimals, units)

Response

Corrected throughout: Decimal format standardized (e.g., 41.15), and Units standardized (kg, g, %, °C)

• The morbidity table (Table 11) appears to contain an error: the parameter heading states “Mortality (%)” instead of “Morbidity (%)”.

Response

Thank you for identifying this error. Following the reanalysis of the morbidity data using an appropriate binomial logistic regression approach, Table 11 (Line 469) has been fully updated to reflect the revised results. The corrected table now accurately presents morbidity outcomes and is consistent with the statistical methods described in the revised Methods section (Lines 271-288).

• In the feed composition section, it would be useful to provide metabolizable energy for all diet phases and clearer feed formulation details.

Response

Thank you for this helpful suggestion. The feed composition section has been revised and expanded to improve clarity and reproducibility.

Specifically:

Metabolizable energy (ME) values for all dietary phases (starter, grower, and finisher) have now been included (Line 171).

The ingredient composition and proportions of each diet have been clearly presented.

Additional details on feed formulation, including the inclusion of the toxin binder, have been clarified to ensure transparency.

These revisions provide a more comprehensive description of the diets used and enhance the reproducibility of the study.

• References formatting

Response

All references have been revised to match PLOS ONE style (Lines 629-735).

• Figures and captions

Response

All figures: Properly labeled, Referenced in text, and Captions improved for clarity

EDITORIAL REQUIREMENTS

Funding statement correction

Response

Funding-related text has been removed from Acknowledgments.

Updated statement (to include in cover letter):

“The authors received no specific funding for this work.”

Data availability

Response

Confirmed:

• All raw data supporting results are included as Supporting Information

Statement updated accordingly.

We believe that the manuscript has been substantially improved in response to all reviewers and academic editor comments. We respectfully submit the revised manuscript for reconsideration and hope it is now suitable for publication in PLOS ONE.

Sincerely,

Dennis Owusu Acheampong

(on behalf of all authors)

Attachment Submitted filename: Response to Reviewers.docx

# Peer review: Decision Letter 1

**DOI:** 10.1371/journal.pone.0351927.r003

**Authors:** Lamiaa Mostafa Radwan

18 May 2026

PONE-D-26-01344R1

Integrated Assessment of Growth Performance, Carcass Characteristics, and Economic Efficiency of Three Broiler Strains Raised Under Humid-Tropical Conditions

PLOS One

Dear Dr. ACHEAMPONG,

Thank you for submitting your manuscript to PLOS ONE. After careful consideration, we feel that it has merit but does not fully meet PLOS ONE’s publication criteria as it currently stands. Therefore, we invite you to submit a revised version of the manuscript that addresses the points raised during the review process.

==============================

Dear Dr., DENNIS OWUSU ACHEAMPONG, MPHIL.

Thank you for submitting your manuscript to PLOS ONE. After careful consideration, we have decided that your manuscript needs Mainor Revision.

Kind regards,

Prof. Lamiaa Mostafa Radwan, Ph.D.

Academic Editor

PLOS ONE

**Reviewer1**

The manuscript has undergone substantial changes and all my comments have been incorporated into the text. I recommend accepting the manuscript.

**Reviewer2**

I observed that the authors did not include the markings in the manuscript indicating where the corrections requested in the previous review were made. Therefore, it was not possible to clearly identify the modifications performed.

Thus, I am returning the manuscript so that the authors can resubmit the files with the corrections properly indicated in the document.

Sincerely.

==============================

Please submit your revised manuscript by Jul 02 2026 11:59PM. If you will need more time than this to complete your revisions, please reply to this message or contact the journal office at plosone@plos.org. When you're ready to submit your revision, log on to https://www.editorialmanager.com/pone/ and select the 'Submissions Needing Revision' folder to locate your manuscript file.

Please include the following items when submitting your revised manuscript:

- A letter that responds to each point raised by the academic editor and reviewer(s). You should upload this letter as a separate file labeled 'Response to Reviewers'.
- A marked-up copy of your manuscript that highlights changes made to the original version. You should upload this as a separate file labeled 'Revised Manuscript with Track Changes'.
- An unmarked version of your revised paper without tracked changes. You should upload this as a separate file labeled 'Manuscript'.

If you would like to make changes to your financial disclosure, please include your updated statement in your cover letter. Guidelines for resubmitting your figure files are available below the reviewer comments at the end of this letter.

If applicable, we recommend that you deposit your laboratory protocols in protocols.io to enhance the reproducibility of your results. Protocols.io assigns your protocol its own identifier (DOI) so that it can be cited independently in the future. For instructions see: https://journals.plos.org/plosone/s/submission-guidelines#loc-laboratory-protocols. Additionally, PLOS ONE offers an option for publishing peer-reviewed Lab Protocol articles, which describe protocols hosted on protocols.io. Read more information on sharing protocols at https://plos.org/protocols?utm_medium=editorial-email&utm_source=authorletters&utm_campaign=protocols.

As the corresponding author, your ORCID iD is verified in the submission system and will appear in the published article. PLOS supports the use of ORCID, and we encourage all coauthors to register for an ORCID iD and use it as well. Please encourage your coauthors to verify their ORCID iD within the submission system before final acceptance, as unverified ORCID iDs will not appear in the published article. *Only*   the individual author can complete the verification step; PLOS staff *cannot*   verify ORCID iDs on behalf of authors.

We look forward to receiving your revised manuscript.

Kind regards,

Lamiaa Mostafa Radwan, Ph.D.

Academic Editor

PLOS One

Journal Requirements:

1. If the reviewer comments include a recommendation to cite specific previously published works, please review and evaluate these publications to determine whether they are relevant and should be cited. There is no requirement to cite these works unless the editor has indicated otherwise.

2. Please review your reference list to ensure that it is complete and correct. If you have cited papers that have been retracted, please include the rationale for doing so in the manuscript text, or remove these references and replace them with relevant current references. Any changes to the reference list should be mentioned in the rebuttal letter that accompanies your revised manuscript. If you need to cite a retracted article, indicate the article’s retracted status in the References list and also include a citation and full reference for the retraction notice.

**Additional Editor Comments:**

Dear Dr., DENNIS OWUSU ACHEAMPONG, MPHIL.

Thank you for submitting your manuscript to PLOS ONE. After careful consideration, we have decided that your manuscript needs Mainor Revision.

Kind regards,

Prof. Lamiaa Mostafa Radwan, Ph.D.

Academic Editor

PLOS ONE

Reviewer1

The manuscript has undergone substantial changes and all my comments have been incorporated into the text. I recommend accepting the manuscript.

Reviewer2

I observed that the authors did not include the markings in the manuscript indicating where the corrections requested in the previous review were made. Therefore, it was not possible to clearly identify the modifications performed.

Thus, I am returning the manuscript so that the authors can resubmit the files with the corrections properly indicated in the document.

Sincerely.

[Note: HTML markup is below. Please do not edit.]

Reviewers' comments:

Reviewer's Responses to Questions

**Comments to the Author**

1. If the authors have adequately addressed your comments raised in a previous round of review and you feel that this manuscript is now acceptable for publication, you may indicate that here to bypass the “Comments to the Author” section, enter your conflict of interest statement in the “Confidential to Editor” section, and submit your "Accept" recommendation.

Reviewer #1: All comments have been addressed

Reviewer #2: (No Response)

**********

2. Is the manuscript technically sound, and do the data support the conclusions?

The manuscript must describe a technically sound piece of scientific research with data that supports the conclusions. Experiments must have been conducted rigorously, with appropriate controls, replication, and sample sizes. The conclusions must be drawn appropriately based on the data presented.

Reviewer #1: Yes

Reviewer #2: Yes

**********

3. Has the statistical analysis been performed appropriately and rigorously?

Reviewer #1: Yes

Reviewer #2: Yes

**********

4. Have the authors made all data underlying the findings in their manuscript fully available?

The PLOS Data policy requires authors to make all data underlying the findings described in their manuscript fully available without restriction, with rare exception (please refer to the Data Availability Statement in the manuscript PDF file). The data should be provided as part of the manuscript or its supporting information, or deposited to a public repository. For example, in addition to summary statistics, the data points behind means, medians and variance measures should be available. If there are restrictions on publicly sharing data—e.g. participant privacy or use of data from a third party—those must be specified.

Reviewer #1: Yes

Reviewer #2: Yes

**********

5. Is the manuscript presented in an intelligible fashion and written in standard English?

PLOS ONE does not copyedit accepted manuscripts, so the language in submitted articles must be clear, correct, and unambiguous. Any typographical or grammatical errors should be corrected at revision, so please note any specific errors here.

Reviewer #1: Yes

Reviewer #2: Yes

**********

6. Review Comments to the Author

Please use the space provided to explain your answers to the questions above. You may also include additional comments for the author, including concerns about dual publication, research ethics, or publication ethics. (Please upload your review as an attachment if it exceeds 20,000 characters)

Reviewer #1: The manuscript has undergone substantial changes and all my comments have been incorporated into the text. I recommend accepting the manuscript.

Reviewer #2: I observed that the authors did not include the markings in the manuscript indicating where the corrections requested in the previous review were made. Therefore, it was not possible to clearly identify the modifications performed.

Thus, I am returning the manuscript so that the authors can resubmit the files with the corrections properly indicated in the document.

Sincerely.

**********

7. PLOS authors have the option to publish the peer review history of their article (what does this mean?). If published, this will include your full peer review and any attached files.

If you choose “no”, your identity will remain anonymous but your review may still be made public.

**Do you want your identity to be public for this peer review?**   For information about this choice, including consent withdrawal, please see our Privacy Policy.

Reviewer #1: **Yes:**   František Zigo

Reviewer #2: **Yes:**   Dr. Manish K. Singh

**********

[NOTE: If reviewer comments were submitted as an attachment file, they will be attached to this email and accessible via the submission site. Please log into your account, locate the manuscript record, and check for the action link "View Attachments". If this link does not appear, there are no attachment files.]

To ensure your figures meet our technical requirements, please review our figure guidelines: https://journals.plos.org/plosone/s/figures

You may also use PLOS’s free figure tool, NAAS, to help you prepare publication quality figures: https://journals.plos.org/plosone/s/figures#loc-tools-for-figure-preparation.

NAAS will assess whether your figures meet our technical requirements by comparing each figure against our figure specifications.

Attachment Submitted filename: Final Corrections.docx

# Author comment: Author response to Decision Letter 2

**DOI:** 10.1371/journal.pone.0351927.r004

28 May 2026

Response to Reviewer 2

Comment:

“I observed that the authors did not include the markings in the manuscript indicating where the corrections requested in the previous review were made. Therefore, it was not possible to clearly identify the modifications performed.

Thus, I am returning the manuscript so that the authors can resubmit the files with the corrections properly indicated in the document.”

Response:

We sincerely apologize for the inconvenience caused by the previously submitted revision files. In the earlier submission, the tracked revisions and markings were not properly visible in the uploaded manuscript file. To address this issue, we have now carefully prepared and uploaded a fully marked revised manuscript with all modifications clearly indicated using Microsoft Word “Track Changes” (All Markup view enabled).

Additionally, revised sections have been clearly highlighted throughout the document to facilitate evaluation of the changes made in response to the reviewers’ comments. We appreciate the reviewer’s observation and thank them for helping us improve the clarity of the revised submission.

Attachment Submitted filename: Response_to_Reviewers_auresp_2.docx

# Peer review: Decision Letter 2

**DOI:** 10.1371/journal.pone.0351927.r005

**Authors:** Lamiaa Mostafa Radwan

3 Jun 2026

Integrated Assessment of Growth Performance, Carcass Characteristics, and Economic Efficiency of Three Broiler Strains Raised Under Humid-Tropical Conditions

PONE-D-26-01344R2

Dear Dr. DENNIS OWUSU ACHEAMPONG, MPHIL,

We’re pleased to inform you that your manuscript has been judged scientifically suitable for publication and will be formally accepted for publication once it meets all outstanding technical requirements.

Within one week, you’ll receive an e-mail detailing the required amendments. When these have been addressed, you’ll receive a formal acceptance letter and your manuscript will be scheduled for publication.

An invoice will be generated when your article is formally accepted. Please note, if your institution has a publishing partnership with PLOS and your article meets the relevant criteria, all or part of your publication costs will be covered. Please make sure your user information is up-to-date by logging into Editorial Manager at Editorial Manager® and clicking the ‘Update My Information' link at the top of the page. For questions related to billing, please contact billing support.

If your institution or institutions have a press office, please notify them about your upcoming paper to help maximize its impact. If they’ll be preparing press materials, please inform our press team as soon as possible -- no later than 48 hours after receiving the formal acceptance. Your manuscript will remain under strict press embargo until 2 pm Eastern Time on the date of publication. For more information, please contact onepress@plos.org.

Kind regards,

Lamiaa Mostafa Radwan, Ph.D.

Academic Editor

PLOS One

Additional Editor Comments (optional):

Dear Dr., DENNIS OWUSU ACHEAMPONG, MPHIL.

I am pleased to inform you that the manuscript has been accepted for publication.

Kind regards,

Prof. Lamiaa Mostafa Radwan, Ph.D.

Academic Editor

PLOS ONE

Reviewer 1

The authors incorporated all my comments into the text, which led to its improvement. I recommend accepting the manuscript in its present form.

Reviewer 2

I appreciate your efforts in revising the manuscript and have no further comments at this stage.

Best regards,

Reviewers' comments:

Reviewer's Responses to Questions

**Comments to the Author**

1. If the authors have adequately addressed your comments raised in a previous round of review and you feel that this manuscript is now acceptable for publication, you may indicate that here to bypass the “Comments to the Author” section, enter your conflict of interest statement in the “Confidential to Editor” section, and submit your "Accept" recommendation.

Reviewer #1: All comments have been addressed

Reviewer #2: All comments have been addressed

**********

2. Is the manuscript technically sound, and do the data support the conclusions?

The manuscript must describe a technically sound piece of scientific research with data that supports the conclusions. Experiments must have been conducted rigorously, with appropriate controls, replication, and sample sizes. The conclusions must be drawn appropriately based on the data presented.

Reviewer #1: Yes

Reviewer #2: Yes

**********

3. Has the statistical analysis been performed appropriately and rigorously?

Reviewer #1: Yes

Reviewer #2: (No Response)

**********

4. Have the authors made all data underlying the findings in their manuscript fully available?

The PLOS Data policy requires authors to make all data underlying the findings described in their manuscript fully available without restriction, with rare exception (please refer to the Data Availability Statement in the manuscript PDF file). The data should be provided as part of the manuscript or its supporting information, or deposited to a public repository. For example, in addition to summary statistics, the data points behind means, medians and variance measures should be available. If there are restrictions on publicly sharing data—e.g. participant privacy or use of data from a third party—those must be specified.

Reviewer #1: Yes

Reviewer #2: Yes

**********

5. Is the manuscript presented in an intelligible fashion and written in standard English?

PLOS ONE does not copyedit accepted manuscripts, so the language in submitted articles must be clear, correct, and unambiguous. Any typographical or grammatical errors should be corrected at revision, so please note any specific errors here.

Reviewer #1: Yes

Reviewer #2: Yes

**********

6. Review Comments to the Author

Please use the space provided to explain your answers to the questions above. You may also include additional comments for the author, including concerns about dual publication, research ethics, or publication ethics. (Please upload your review as an attachment if it exceeds 20,000 characters)

Reviewer #1: The authors incorporated all my comments into the text, which led to its improvement. I recommend accepting the manuscript in its present form.

Reviewer #2: I appreciate your efforts in revising the manuscript and have no further comments at this stage.

Best regards,

(MKS)

**********

7. PLOS authors have the option to publish the peer review history of their article (what does this mean?). If published, this will include your full peer review and any attached files.

If you choose “no”, your identity will remain anonymous but your review may still be made public.

**Do you want your identity to be public for this peer review?**   For information about this choice, including consent withdrawal, please see our Privacy Policy.

Reviewer #1: **Yes:**   František Zigo

Reviewer #2: **Yes:**   Dr. Manish K. Singh (Ph.D. Poultry Science)

**********

Attachment Submitted filename: Response to Reviewers.doc

# Editor report: Acceptance letter

**DOI:** 10.1371/journal.pone.0351927.r006

**Authors:** Lamiaa Mostafa Radwan

PONE-D-26-01344R2

PLOS One

Dear Dr. ACHEAMPONG,

I'm pleased to inform you that your manuscript has been deemed suitable for publication in PLOS One. Congratulations! Your manuscript is now being handed over to our production team.

At this stage, our production department will prepare your paper for publication. This includes ensuring the following:

* All references, tables, and figures are properly cited

* All relevant supporting information is included in the manuscript submission,

* There are no issues that prevent the paper from being properly typeset

You will receive further instructions from the production team, including instructions on how to review your proof when it is ready. Please keep in mind that we are working through a large volume of accepted articles, so please give us a few days to review your paper and let you know the next and final steps.

Lastly, if your institution or institutions have a press office, please let them know about your upcoming paper now to help maximize its impact. If they'll be preparing press materials, please inform our press team within the next 48 hours. Your manuscript will remain under strict press embargo until 2 pm Eastern Time on the date of publication. For more information, please contact onepress@plos.org.

You will receive an invoice from PLOS for your publication fee after your manuscript has reached the completed accept phase. If you receive an email requesting payment before acceptance or for any other service, this may be a phishing scheme. Learn how to identify phishing emails and protect your accounts at https://explore.plos.org/phishing.

If we can help with anything else, please email us at customercare@plos.org.

Thank you for submitting your work to PLOS ONE and supporting open access.

Kind regards,

PLOS ONE Editorial Office Staff

on behalf of

Prof. Dr. Lamiaa Mostafa Radwan

Academic Editor

PLOS One
